# Barley HvHMA1 Is a Heavy Metal Pump Involved in Mobilizing Organellar Zn and Cu and Plays a Role in Metal Loading into Grains

**DOI:** 10.1371/journal.pone.0049027

**Published:** 2012-11-14

**Authors:** Maria Dalgaard Mikkelsen, Pai Pedas, Michaela Schiller, Eva Vincze, Rebecca F. Mills, Søren Borg, Annette Møller, Jan K. Schjoerring, Lorraine E. Williams, Lone Baekgaard, Preben Bach Holm, Michael G. Palmgren

**Affiliations:** 1 Centre for Membrane Pumps in Cells and Disease (PUMPKIN), Danish National Research Foundation, Frederiksberg, Denmark; 2 Department of Plant and Environmental Sciences, University of Copenhagen, Frederiksberg, Denmark; 3 Department of Molecular Biology and Genetics, Research Centre Flakkebjerg, Aarhus University, Slagelse, Denmark; 4 Centre for Biological Sciences, University of Southampton, Southampton, Hampshire, United Kingdom; University of Cambridge, United Kingdom

## Abstract

Heavy metal transporters belonging to the P_1B_-ATPase subfamily of P-type ATPases are key players in cellular heavy metal homeostasis. Heavy metal transporters belonging to the P_1B_-ATPase subfamily of P-type ATPases are key players in cellular heavy metal homeostasis. In this study we investigated the properties of HvHMA1, which is a barley orthologue of Arabidopsis thaliana AtHMA1 localized to the chloroplast envelope. HvHMA1 was localized to the periphery of chloroplast of leaves and in intracellular compartments of grain aleurone cells. HvHMA1 expression was significantly higher in grains compared to leaves. In leaves, HvHMA1 expression was moderately induced by Zn deficiency, but reduced by toxic levels of Zn, Cu and Cd. Isolated barley chloroplasts exported Zn and Cu when supplied with Mg-ATP and this transport was inhibited by the AtHMA1 inhibitor thapsigargin. Down-regulation of HvHMA1 by RNA interference did not have an effect on foliar Zn and Cu contents but resulted in a significant increase in grain Zn and Cu content. Heterologous expression of HvHMA1 in heavy metal-sensitive yeast strains increased their sensitivity to Zn, but also to Cu, Co, Cd, Ca, Mn, and Fe. Based on these results, we suggest that HvHMA1 is a broad-specificity exporter of metals from chloroplasts and serve as a scavenging mechanism for mobilizing plastid Zn and Cu when cells become deficient in these elements. In grains, HvHMA1 might be involved in mobilizing Zn and Cu from the aleurone cells during grain filling and germination.

## Introduction

In all cells, Zn and Cu are essential heavy metal micronutrients although in excess they can be toxic [1]–[5]. They function as cofactors, either as structural stabilizers such as transcription factors, or as functional components of proteins, like in the active sites of enzymes [3], [6], [7]. Zn and Cu are therefore involved in a wide range of processes, ranging from initiation of DNA transcription to making photosynthesis possible [6]–[9]. In chloroplasts, Cu plays a vital role in photosynthesis through incorporation into plastocyanin, while both Cu and Zn are used as cofactors in Cu/Zn superoxide dismutase (SOD) [10]–[11].

Because Zn and Cu are essential in all cells but toxic in excess, their transport and homeostasis are tightly regulated [12]–[14]. In plants, these heavy metals are taken up from the soil through the roots and subsequently exported from xylem parenchyma cells into xylem vessels responsible for long distance transport to the shoot [15]–[17]. During senescence of cereal leaves, Zn and Cu are mobilized to the developing grain [17]–[20]. Zn is important for germination, as seeds of low Zn content show poor germination and seedling development [21]. In grains during grain filling, Zn and Cu accumulate in the embryo and the aleurone layer, while in addition large amounts accumulate in the pericarp, the maternally produced tissue surrounding the seed [21]–[23]. Transport from the pericarp to the inner grain through the highly specified transfer cells in the maternal/filial grain barrier is thought to be a limiting step in heavy metal loading into the grain, although knowledge in this area is scarce [17], [21], [24]. Cu and Zn have to exit the maternal cells before grain loading, as these cells are not in symplastic continuum with the grain filial cells [24], [25]. Furthermore, export of positively charged heavy metal ions from maternal cells has to be active in order to overcome the positive-outside membrane potential created by plasma membrane H^+^-ATPases [17], [24], [25].

Heavy metal pumps belong to the super-family of P-type ATPase pumps, named so because each catalytic cycle is initiated by phosphorylation of a conserved aspartic acid residue [26], [27]. Five major P-type ATPase sub-families (P_1_ to P_5_) pump different cations over membranes, except for the P_4_ sub-family that has been implicated in phospholipid flipping [26]–[28]. P_1B_-ATPases are involved in heavy metal homeostasis in organisms ranging from bacteria to higher plants and humans [26], [29]. These pumps are involved in heavy metal compartmentalization, chelation, and cell export to ensure that heavy metal concentrations remain in a narrow range to meet the need of the cell and organism without causing toxicity [29], [30]. Common features of P_1B_-ATPases include the CPX/SPC domain in transmembrane domain 6 involved in metal-binding during transport as well as N- and C-terminal metal binding domains (N- and C-MBDs), which may be involved in regulation of activity [29]–[35].

The model dicotyledonous plant *Arabidopsis thaliana* contains eight P_1B_-ATPases, which can be divided into two groups according to their putative substrate specificity [29], [36]. AtHMA5 to 8 belong to Group 1 and are predicted to transport Cu/Ag, while AtHMA1 to 4 belong to Group 2 and are predicted to transport Zn/Co/Cd/Pb [29], [36]. Among the *A. thaliana* P_1B_-ATPases, AtHMA2 and AtHMA4 show redundant function in cellular export of Zn and Cd in plant vascular tissues to ensure subsequent xylem loading and transport to the shoot [16], [35], [37]. When both genes have been knocked out, *A. thaliana* plants exhibit a strong Zn nutritional deficient phenotype [16], [35]. This phenotype has recently been shown to be suppressed by the barley orthologue HvHMA2, suggesting a similar role of HvHMA2 as AtHMA2 and 4 [38]. The *A. thaliana* AtHMA7 has been shown to actively pump Cu into the post-Golgi compartment, a prerequisite for maturation of the ethylene receptor [39], [40], while AtHMA6, localized to the chloroplast inner envelope membrane, delivers Cu to Cu/Zn-SOD or further to AtHMA8 that is localized to the thylakoid membrane where it supplies Cu to plastocyanin [41], [42].

AtHMA1 is localized to the chloroplast inner envelope membrane [43], [44]. Conflicting results have been reported in the literature with respect to the ion specificity and function of this pump. According to one model, AtHMA1 transports Cu into the stroma of chloroplasts [44] whereas another model predicts that AtHMA1 is involved in the export of Zn from chloroplasts [43]. AtHMA1 has also been implicated in the transport of Ca [45]. *Athma1* knock-out plants show a high light phenotype, the severity of which depends on individual plants, displaying everything from wild-type phenotype to severe dwarfism and variegated whitening of leaves [44]. The whitening of leaves was suggested to be caused by chloroplast Cu deficiency, since chloroplasts of *athma1* knockout plants are reduced in Cu content and showed a decreased Cu-stimulated ATPase activity compared to wild-type [44]. Due to lower apparent activity of SOD in *Athma1* knockout plants compared to wild-type, it has been suggested that AtHMA1 feeds the stromal Cu/Zn-SOD with Cu [44], [46]. However, the high light phenotype of *Athma1* knockout plants could not be rescued by addition of Cu [44]. In contrast, Kim *et al.*, (2009) [43] found that the chloroplastic Zn content of *Athma1* knockout plants increased on Zn containing media, while the Cu content was unaffected. Furthermore, *Athma1* knockout plants are more sensitive than wild-type to elevated Zn, an effect which is unrelated to light conditions [43]. It was therefore suggested that AtHMA1 exports Zn from the chloroplast and serves as a detoxification mechanism when Zn levels become toxic [43].

In this work, we have cloned *HvHMA1*, an orthologue of *AtHMA1* from the monocotyledonous cereal *Hordeum vulgare*. We show that HvHMA1 is a broad-specificity pump that can export Zn and Cu from chloroplasts and which is moderately up-regulated in response to Zn deficiency. We propose that HvHMA1 might serve as a scavenging mechanism in remobilizing plastid Zn and Cu when required elsewhere in the cell. Furthermore we suggest that HvHMA1, which is highly expressed in the endosperm, is involved in Zn and Cu homeostasis during grain filling, a function that is important for controlling the total Zn and Cu content in mature grains.

## Materials and Methods

### Cloning of *HvHMA1*

Using information from EST sequence (AV913537) the 3′ end of the *HvHMA1* cDNA sequence (bp 435–2487) was cloned by polymerase chain reaction (PCR) from a barley root cDNA library [47] (Table S1 for primer information). Sequence information of the 5′ end of *HvHMA1* was achieved by screening a Stratagene barley genomic phage library (cultivar Igrid). Using a *HvHMA1* specific fragment, eight *HvHMA1* clones were isolated and sequenced, and the *HvHMA1* genomic fragment of bp 1–435 was obtained. This fragment contained no introns, as verified by comparing the *HvHMA1* sequence with the full-length cDNA clone from rice (*OsHMA1*, AK100055) resulting in the full-length cDNA sequence of *HvHMA1* (accession number FR873736). A 1.516 bp promoter sequence was furthermore cloned from the phage library by employing PCR (primer sequences are listed in Table S1).

### Yeast Constructs and Complementation Experiments

For heterologous expression in the yeast *Saccharomyces cerevisiae*, full-length *HvHMA1* was amplified by PCR, introduced into pJET1/blunt (http://www.fermentas.com), sequenced, and introduced into the pYES2 vector by restriction enzymes and ligation. In this vector, *HvHMA1* is under control of the galactose inducible promoter of *GAL1*. The following modified constructs were made: i) A construct encoding HvHMA1 where the essential Asp457 residue has been substituted by Asn (*Hvhma1*). ii) A construct encoding HvHMA1 where the putative chloroplastic target peptide, including 50 amino acid residues, had been deleted (*Hvhma1Δ50*). iii) A construct encoding HvHMA1 where the 97 amino acid residue long N-terminal domain had been deleted (*Hvhma1Δ97*). iv) A construct containing the N-terminal 117 amino acid residues of HvHMA1 (*Hvhma1Nt*).

Several *S. cerevisiae* mutant strains (Table S2) were used for gene expression and for a positive control transformed with the empty vector (pYES2). Yeast cells were transformed as previously described [48]. Transformed yeast cells were used for drop test experiments for measuring metal tolerance of yeast expressing *HvHMA1* and mutants. Yeast cells were diluted in H_2_O to OD_600_ = 0.5 and 0.05 and spotted on minimal media containing 2% (w/v) galactose (Gal), 2% (w/v) bacto-agar, 0.7% (w/v) yeast nitrogen base (YNB), 20 µg/ml His, 30 µg/ml Met, 30 µg/ml Leu, 30 µg/ml Ade for K616 and metals as indicated. Plates were incubated at 30°C for 3–5 days.

### HMA1p-HMA1-GFP Construct

The *HvHMA1* promoter in front of HvHMA1 with a 3′ GFP fusion was cloned into the Gateway system (Invitrogen, Life Technologies Corporation). The HvHMA1p-HvHMA1-GFP construct had been made by introducing the promoter of *HvHMA1* into the Gateway vector pMDC32 [49] replacing the 2×35S promoter by restriction enzymes and ligation. GFP was amplified from pMDC85 and inserted into the vector by restriction enzymes and ligation (primer sequences are listed in Table S1). In the last step *HvHMA1* cDNA without the stop-codon was inserted by an LR reaction.

### HvHMA1-RNAi Construct

A DNA sequence of 255 bp (Figure S6) covering bp 2108–2362 in *HvHMA1* was made based on EST's. Four oligonucleotides were made (Table S1) and put together by overlapping PCR. The DNA fragment was sequenced and then inserted into the RNAi hairpin Gateway vector pSTARGATE (CSIRO: hairpin RNAi vectors for plants). The RNAi construct of *HvHMA1* was cloned, before the cDNA sequence was obtained, and thus three base substitutions are present compared to the cloned *HvHMA1* sequence. The RNAi sequence was predicted to be unique for *HvHMA1* when BLASTed against ESTs from barley as well as against the rice sequenced genome.

### Barley Material and Growth Conditions

Barley plants cv. Golden Promise for *Agrobacterium*-mediated transformations were grown as previously described [50]. Agrobacterium-mediated transformation of premature barley embryo cells was carried out using the hygromycin resistance gene as selectable marker [51], [52] with additional modifications as described by Carciofi *et al.* (2011) [53]. For transformation of barley, *Agrobacterium tumefaciens* strain (AGL0) [54] was transformed with *HvHMA1* RNAi or with HvHMA1-GFP under control of the *HvHMA1* promoter.

Barley cv. Golden Promise grains were surface sterilized and sown in vermiculite. One week of germination, seedlings were after transferred to a hydroponic system for two or three weeks of growth prior to the start of the treatment, as described previously [55]. For hydroponic analysis under high light conditions barley was grown in 16 hours light and 8 hours darkness for one week after germination in vermiculite in the green house. The light intensity under high light conditions was ∼1000 µmoles m^−2^ s^−1^.

Heavy metal treatments of barley plants in hydroponics were done with three independent biological replicates per treatment, where each replicate consisted of one bucket with 16 barley seedlings. The Zn treatments started two weeks after germination and consisted of fresh basic nutrient solution without Zn but supplied with 125 nM, 10 µM, 100 µM and 1000 µM ZnCl_2_. Treatments involving exposure to Cu or Cd started 3 weeks after germination by addition of 0.4 µM, 5 µM, 50 µM and 500 µM CuSO_4_ or 5 µM, 10 µM and 20 µM CdCl_2_ to Cu-free or standard basic nutrient solution, respectively. Leaves were harvested 24 and 48 hours after treatment start. Leaves from each replicate were cut, homogenized and divided into two samples; one sample for RNA extraction and RT-qPCR measurement. To induce Cu and Zn deficiency, barley was grown in hydroponics for four weeks without addition of Cu or Zn, respectively, to the basic nutrient solution. Five of the youngest fully developed leaves were harvested in each treatment, including a control treatment containing 0.7 µM Zn and 0.8 µM Cu, with three independent biological replicates per treatment.

To study barley seedlings during germination, barley grains were surface sterilised and sown in vermiculite. Shoots and roots were harvested and analysed by qRT-PCR (three replicates of 14–26 plants each) at 3, 5, 7 and 10 days after germination start, while whole seedlings were harvested at 2, 3, 4, 5, 7 and 10 days after germination start. Barley was grown to maturity in soil for harvest of developing grains at 14, 25 and 35 days after pollination (DAP) for qRT-PCR with four biological replicates per time point. All samples for RT-qPCR analysis were frozen immediately in liquid nitrogen and stored at −80°C until RNA extraction.

### *A. thaliana* Material and Growth Conditions

*A. thaliana* ‘Columbia-8’ wild-type, *Athma1* insertion mutants (*Athma1-1* and *Athma1-2* described in Kim *et al.* (2009) [43]) and 35S *HvHMA1::Athma1-2* seedlings were sterilized in 10% (v/v) bleach for 20 min and then rinsed five times with sterile water. Seeds were inoculated onto plates containing 0.8% (w/v) agarose (Melford), 1% (w/v) sucrose, and one-half-strength (0.5) Murashige and Skoog medium [56] as previously described [57]. Seeds were then incubated in the dark at 4°C for 48 h prior to transfer to a controlled-environment cabinet and exposed to a constant high light (300 µmol m^−2^ s^−1^) or low light (72 µmol m−2 s^−1^) regime at 23°C with the plates incubated vertically.

### Isolation of RNA, Synthesis of cDNA and Quantitative Gene Expression Analysis

Plant samples were ground in liquid N prior to RNA extraction. Total RNA was isolated from root or leaf samples using the Fast RNA® Pro Green Kit (MP Biomedicals, Solon, OH, USA), followed by TURBO™ DNase (Applied Biosystems, Austin, TX, USA) treatment of 10 µg RNA per sample. Then 3 µg of the DNase treated RNA was converted to cDNA with M-MuLV Reverse Transcriptase (New England BioLabs, Ipswich, MA, USA), oligo-(dT) and random hexamer primers or using SuperScript™ II RT according to manufacturers protocol. The cDNA was diluted 2.5 to 5 times and normalized to get the same cDNA concentration in all samples. For quantification of *HvHMA1* gene expression *HvActin* (TC131547), *HvGAPDH* [58], *HvRNABP* (Z48624.1) or *HvTUBA* (U40042.1) were used as reference genes for normalization (primer sequences are listed in Table S3). cDNA was amplified by RT-qPCR using a Mx3000P™ Real-Time PCR System (Stratagene, La Jolla, CA USA) in a total volume of 20 µl per reaction including: 1–2 µl diluted cDNA, 0.3 to 1.2 µM gene specific primers (Table S3), 1×DyNAmo™ Flash Master Mix and 0.4×ROX™ Passive Reference dye from DyNAmo™ Flash SYBER® Green qPCR Kit (Finnzymes, Espoo, Finland). For expression analysis of wild-type barley, the RT-qPCR programme was set as follows: One cycle at 95°C for 7 minutes, followed by 40 cycles of 95°C for 10 seconds and 60°C for 30 seconds, except for expression analysis of individual tissues (leaf, stem, root, embryo, endosperm and rest), for quantification of *HvHMA1* expression in *HvHMA1-RNAi* barley plants and for confirmation of gDNA insertion (primer sequences are listed in Table S3), where RT-qPCR was carried out in a total volume of 10 µl using 5 µl of Power SYBR Green master mix (Applied Biosystems, Foster City, CA, USA), 500 nM forward and reverse primer, 1 µl of cDNA template, and MilliQ H2O up to 10 µl. PCR was performed in an AB7900HT sequence detection system (Applied Biosystems) programmed with the following thermal profile set-up: one cycle at 50°C for 2 min; one cycle at 95°C for 2 min; 40 cycles at 95°C for 15 s and 60°C for 1 min. A dissociation curve to check specificity of the amplified products was performed in the end of each programme with one cycle at 95°C for 1 min, 60°C for 30 seconds, ramping up to 95°C, followed by 1 minute at 95°C. Three biological replicates of each treatment were included and each reaction was performed in duplicate or triplicate. The Pfaffl equation [59] was applied to calculate the relative expression levels. A standard curve was performed for each primer pair prior to RT-qPCR analysis in order to determine the amplification efficiency required for the Pfaffl equation.

The reference genes used in all quantitative experiments were tested for stability under the different conditions and the most stable reference gene was chosen (primer sequences are listed in Table S3). *HvRNABP* was used as an internal control in the Cu/Zn deficiency and the Zn toxicity experiment. *HvActin* was applied as internal control for *HvHMA1* expression in whole seedlings, leaf, stem, root, embryo, endosperm, rest, as well as in the Cu and Cd toxicity study. *HvGAPDH* was used as internal control in germinating barley shoots. *HvRNABP* was furthermore used as a reference and normalization gene for gDNA and *HvTUBA* was used for RT-qPCR on cDNA from RNAi plants. gDNA was purified from barley leaves and used for RT-qPCR using ubiquitin specific primers (Table S3).

### GFP-Fluorescence

Epidermal strips were made from barley leaves and sections of grains were made in the late milking stage. Preparations were visualized using a Leica TCS SP2/MP confocal laser scanning microscope (Leica Microsystems). GFP was excitated at 488 nm, and emission was detected between 500 and 575 nm. Chlorophyll fluorescence emission was detected between 650 and 705 nm. Transient expression of HvHMA1-GFP in tobacco was recorded as reported previously [28].

### Chloroplast Isolation and Transport Assay

Intact chloroplasts were isolated from seven day old *H. vulgare* plants as previously described [60]. Chloroplasts were washed twice in 400 mM sorbitol, 20 mM Hepes/KOH pH 7.6, 2.5 mM EDTA, 8 mM MgCl_2_. Chloroplasts were incubated 15 min on ice in 1 ml buffer (400 mM sorbitol, 20 mM Hepes/KOH pH 7.6, 8 mM MgCl_2_, ±100 nM thapsigargin). Chloroplasts were incubated two hours at 25°C in light after addition of 0.05 mM CuSO_4_ and 0.05 mM ZnSO_4_ ±3 mM Mg-ATP. Chloroplasts were washed twice in (400 mM sorbitol, 20 mM Hepes/KOH pH 7.6, 2.5 mM EDTA, 8 mM MgCl_2_). Chlorophyll content was determined in the chloroplasts by absorbance measurement at 652 nm in 80% acetone. Chloroplasts were after the assay digested in ultra-pure nitric acid and analyzed by ICP-MS (Agilent 7500ce, Agilent Technologies).

### Elemental Analysis of Plant Material

Prior to analysis by ICP-MS and ICP-OES (Optima 5300 DV, PerkinElmer, USA) plant samples were freeze dried (Christ Alpha 2–4; Martin Christ GmbH) and digested using ultra-pure acids as previously described [55], [61].

### Expression of *HvHMA1* in the *A. thaliana Athma1-2* Mutant

*HvHMA1* cloned in pMDC32 after the 35S promoter was transformed into *Agrobacterium tumefaciens* GV3101 by electroporation. *A. thaliana* (Columbia) *Athma1-2* mutant was transformed using the floral dip method but including a 3 hour pre-induction of vir genes by addition of 100 µM acetosyringone to the culture before dipping [62]. Seeds were obtained and transformants were selected by growing the seeds on plates containing 0.8% (w/v) agar, 0.5× Murashige and Skoog (1962) salt medium (Sigma-Aldrich UK) and 1% sucrose with hygromycin (50 µg/ml). Homozygous T3 plants were used for analysis. Several independent lines were isolated and expression confirmed using RT-PCR. RNA and cDNA were prepared and semi-quantitative PCR was performed as previously described [57]. *AtActin2* was used as control; specific primers used are listed in Table S3.

### Chlorophyll and Fresh Weight Determination of Arabidopsis Seedlings

Fresh-weight and chlorophyll measurements were determined as described previously [57] using seedlings grown on five or six separate plates, each plate having four wild-type seedlings, four *Athma1-2* mutants and four 35S *HvHMA1::Athma1-2* seedlings. Chlorophyll was determined following extraction in N,N-dimethylformamide [63].

## Results and Discussion

### Sequence Analysis of HvHMA1, a Close Orthologue of AtHMA1

It was previously found by sequence analysis of expressed sequence tags (ESTs) that barley contains a P_1B_-ATPase with homology to rice *OsHMA1* and *A. thaliana AtHMA1* [29]. We cloned the full-length *HvHMA1* gene from barley and found that it encodes a polypeptide of 828 amino acid residues sharing 87% identity to rice OsHMA1 and 63% identity to the AtHMA1 sequence. Interestingly, the HvHMA1 protein contains a sequence motif DEFGEHYSK in the transmembrane (TM) domain, which is highly similar to DEFGEQLSK, involved in the very specific binding of the plant metabolite thapsigargin to animal and human SERCA pumps [64], [65], [66] and to AtHMA1 [45]. The N-terminal domain in HvHMA1 contains an amino acid stretch of 17 His residues, where 10 are continuous and the remaining seven are spaced with one or two amino acids in between. His residues in the extended termini of P_1B_-ATPases are implicated in metal binding and regulation of pump activity and turn over [31], [67], [37]. Further, the N-terminal domain of HvHMA1 was predicted by ChloroP (http://www.cbs.dtu.dk/services/ChloroP/) to contain a chloroplast target peptide.

**Figure 1:**
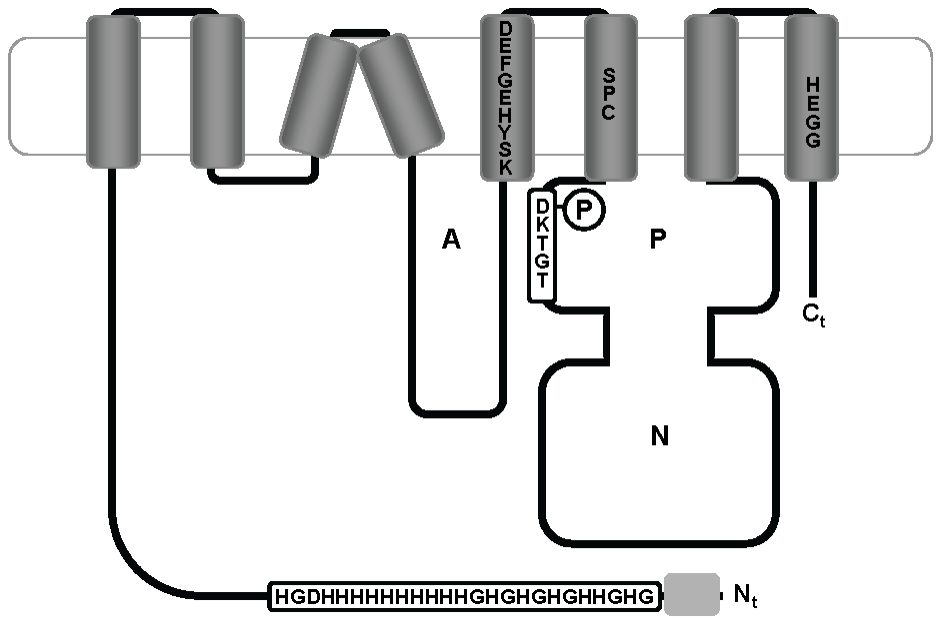
Schematic representation of HvHMA1. HvHMA1 contains the conserved P-type ATPase actuator (A), phosphorylation (P) and nucleotide binding (N) domains. HvHMA1 is here presented with 8 transmembrane segments according to alignment with AtHMA7. HvHMA1 contains an extended N-terminus with a predicted chloroplast target peptide (light gray box) and a long stretch of putative metal-binding histidine residues as indicated. It contains the conserved P-type ATPase phosphorylation sequence DKTGT as well as the P_1B_-ATPase putative metal binding-site SPC and the HEGG motif. Furthermore a putative Tg binding sequence DEFGEHYSK is presented in TM5.

Following analysis by different transmembrane prediction programs, AtHMA1 was predicted to contain only six or seven TM segments, with two or three TM segments preceding the A-domain [43], [44] and the same software obtained comparable results for HvHMA1. However, when aligning the HvHMA1 sequence to P_1B_-ATPases with eight predicted TM segments (Figure S1), it was evident that the third predicted TM segment of HvHMA1 corresponds to TM segments 3 and 4 of AtHMA7. This would suggest that the luminal loop between TM 3 and 4 is deleted in HMA1 and that the third predicted TM segment corresponds to two TM segments arranged in a hairpin structure. According to this interpretation of the sequence, HMA1 has eight TM segments like other P_1B_-ATPases (Figure 1).

### GFP-tagged HvHMA1 Localizes to Chloroplasts of Leaves and the Aleurone Layer of Grains

**Figure 2:**
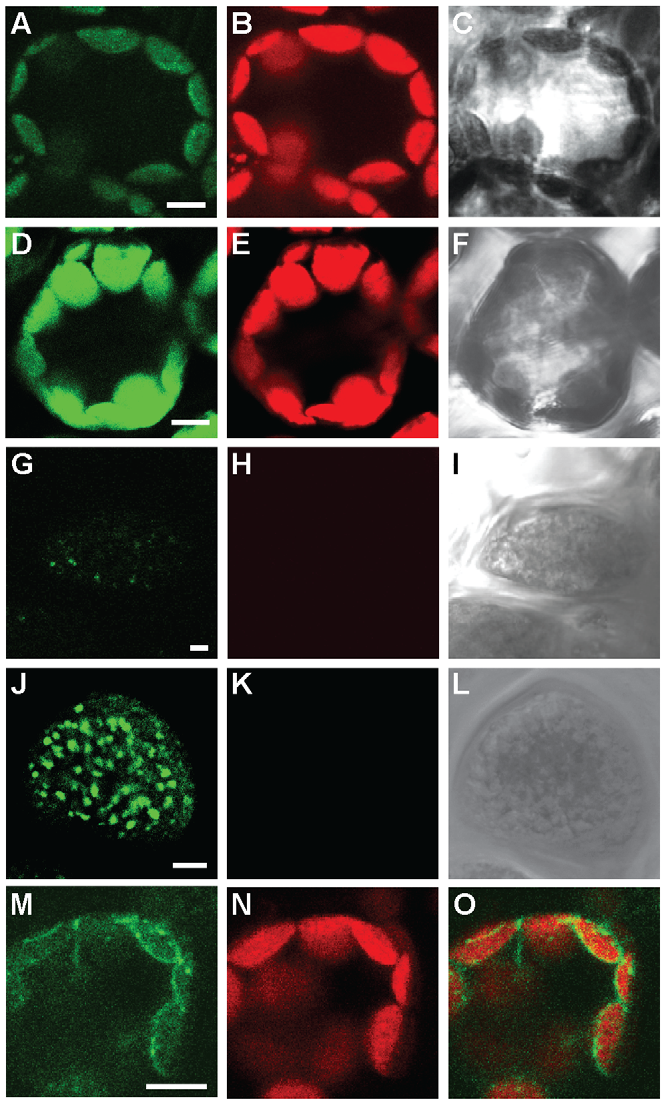
HvHMA1-GFP localizes to chloroplasts of leaves and intracellular compartments of aleurone layer cells of grains. First row (A, B, C) shows a wild-type *H. vulgare* leaf cell, while the second row (D, E, F) shows a transgenic *HvHMA1::GFP H. vulgare* leaf cell. Third row (G, H, I) shows a wild-type aleurone layer cell and forth row (J, K, L) shows a transgenic *H. vulgare* aleurone layer cell expressing *HvHMA1::GFP*. Fifth row (M, N, O) shows a tobacco leaf cell transiently expressing *HvHMA1::GFP* under the control of the HvHMA1 promoter. A), D), G) J) and M) shows emission light at ∼525 nm. B), E), H) K) and N) shows chlorophyll autofluorescence. C), F), I) and L) transmission light images and O) is an overlay of M) and N). Bar is 5 µm.

To confirm a chloroplast localization of HvHMA1, the coding sequence was fused from the 3′ end to *GFP* and the resulting gene construct was stably transformed into barley under expression of the cloned promoter sequence of *HvHMA1*. Strong GFP fluorescence was found in chloroplasts of leaves (Figure 2A–F). Transient expression of *HvHMA1-GFP* in epidermal cells of tobacco revealed that GFP fluorescence is in the periphery of chloroplasts, most likely the chloroplast envelope (Figure 2M–O). GFP localization experiments were supplemented with λ-scans to show that the fluorescence obtained from the GFP expressing cells are in fact from GFP (Figure S2A and S2B). Interestingly, GFP fluorescence was intense in small organellar structures in the aleurone layer cells of barley grains (Figure 2G–L). These compartments were 0.5–0.1 µm in diameter and taking the plastid targeting signal of *HvHMA1* into account, the labelled structures in aleurone cells are likely to represent proplastids although further experimentation is needed to confirm this assumption. Ultrastructural evidence for the presence of proplastids in wheat aleurone cells has been presented by Bechtel *et al.* (1982) [68]. No visible expression of *HvHMA1-GFP* was evident in the starchy endosperm, which has previously been reported to express *HvHMA1* using quantitative real-time RT-PCR [58]. A likely explanation for this discrepancy could be differences in sensitivity between the two techniques.

### *HvHMA1* is Expressed in Grains and Leaves of Barley

**Figure 3:**
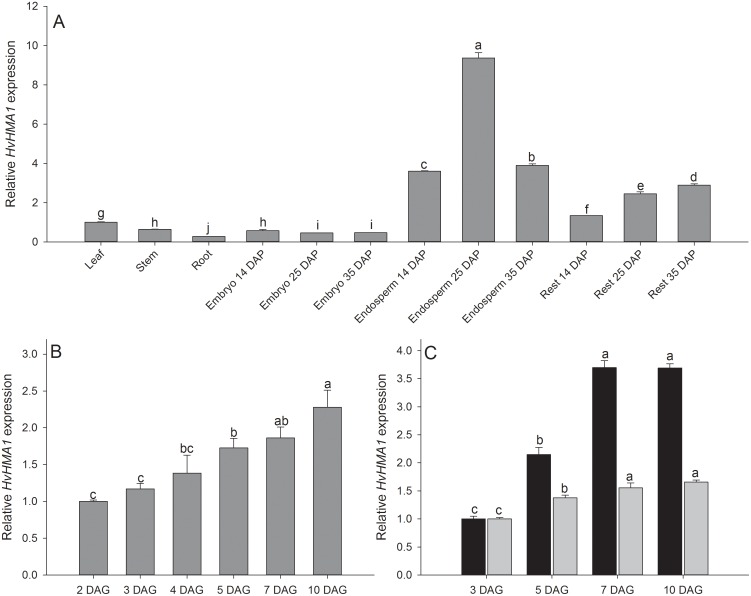
*HvHMA1* is primarily expressed in leaves and grains. A) expression of *HvHMA1* in leaf, stem, root, and grain tissues during grain filling. *HvHMA1* expression is high in leaves and grains, while the expression is lower in stems and roots. In grains during filling *HvHMA1* expression is high in the grain rest (containing the pericarp) and highest in the endosperm, in particular at 25 days after pollination (DAP). B) Expression of *HvHMA1* in whole grains at different days after germination (DAG). The increasing expression during germination is likely caused by up-regulation in shoot expression, which is evident in C) where expression of *HvHMA1* in isolated shoots (black) is up-regulated, while the expression stays low in roots (grey) from germinating seeds. Values with the same letter between treatments and within the same time are not significantly different (P>0.05). Data are the means ± SE (*n* = 3–4).

*HvHMA1* expression was investigated in different tissues by quantitative real-time polymerase chain reaction (RT-qPCR). *HvHMA1* expression was found to be highest in the endosperm of seeds and in leaves, while expression was significantly lower in stems and roots (Figure 3A). In germinating seeds, expression of *HvHMA1* was increased gradually during the first seven days after germination (DAG) (Figure 3B and C). Interestingly, *HvHMA1* was highly expressed in grains during grain filling, which has not previously been described for any P_1B_-ATPase. In grains, the expression of *HvHMA1* was several-fold higher than in leaves and more than doubled at 25 days after pollination (DAP) compared to expression levels at 14 DAP (Figure 3A). Taken together, the results suggest a role of HvHMA1 during grain filling as well as under seed germination.

### *HvHMA1* Expression is Moderately Induced by Zn Deficiency and Decreased by Toxic Levels of Metals

**Figure 4:**
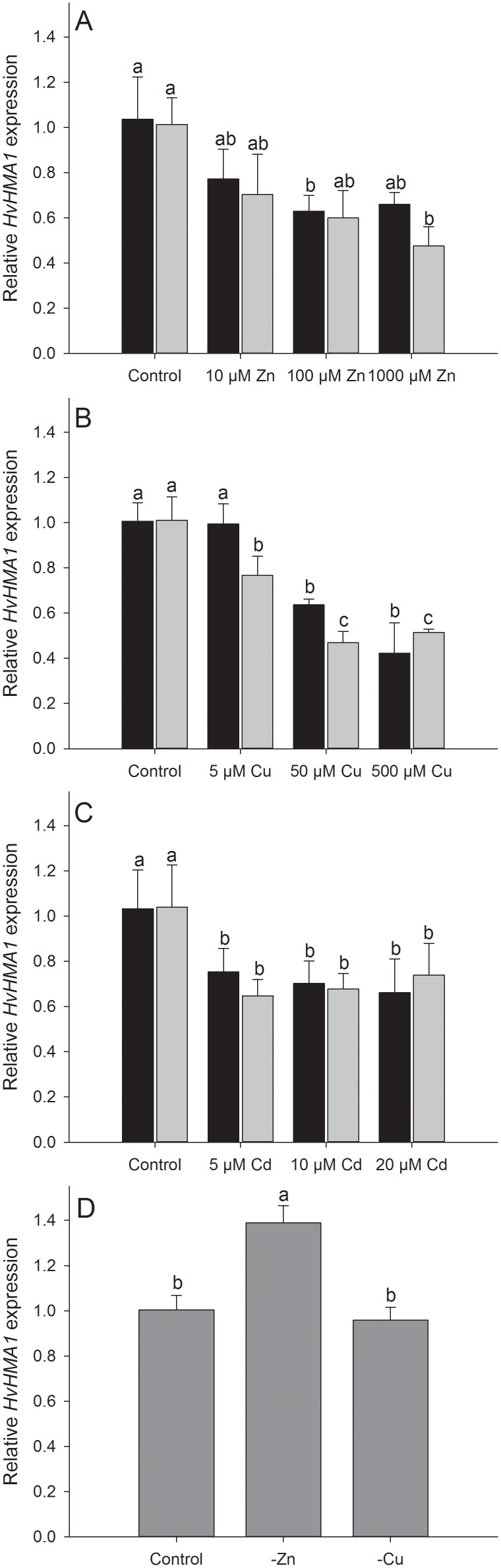
*HvHMA1* expression leaves is down-regulated under metal toxicity and is slightly up-regulated under Zn deficiency. *HvHMA1* expression under A) Zn toxicity after 24 (black) or 48 (grey) hours exposure, B) Cu toxicity after 24 (black) or 48 (grey) hours exposure and C) Cd toxicity after 24 (black) or 48 (black) hours exposure. D) *HvHMA1* expression under control, Zn and Cu deficient conditions. Plants were grown in hydroponics for four weeks. Values with the same letter between treatments and within the same time are not significantly different (P>0.05). Data are the means ± SE (*n* = 3–4). The expression level of *HvHMA1* is slightly increased by Zn deficiency, while it is unchanged under Cu deficiency. When exposed to toxicity of Cd, Zn or Cu the expression level of *HvHMA1* decreases significantly.

The expression level of *HvHMA1* was investigated under different metal stress conditions in hydroponic cultures to determine under which conditions HvHMA1 function may be important. Barley plants exposed to increasing levels of Zn, Cu or Cd for 24 and 48 hours in hydroponic cultures showed a significant decrease in *HvHMA1* expression level in leaves compared to control conditions (Figure 4A–C).

When grown under Cu deficiency, expression of *HvHMA1* in leaves was unchanged compared to plants grown under control conditions (Figure 4D). In contrast, plants grown under Zn deficiency showed a significant increase (approximately 140%) in *HvHMA1* expression compared to control conditions (Figure 4D). These results suggest that HvHMA1 is involved in heavy metal transport under Zn deficiency. It has been suggested that AtHMA1 is involved in detoxification of Zn in chloroplasts, because *Athma1* plants are sensitivity to high Zn [43]. In contrast, we show that expression of *HvHMA1* in barley is significantly down-regulated under toxicity of Zn, but is moderately up-regulated under Zn deficiency. This suggests that HvHMA1 functions under Zn deficiency rather than under toxic levels of this metal.

### Complementation of *A. thaliana Athma1* Knockout Plants by *HvHMA1*

**Figure 5:**
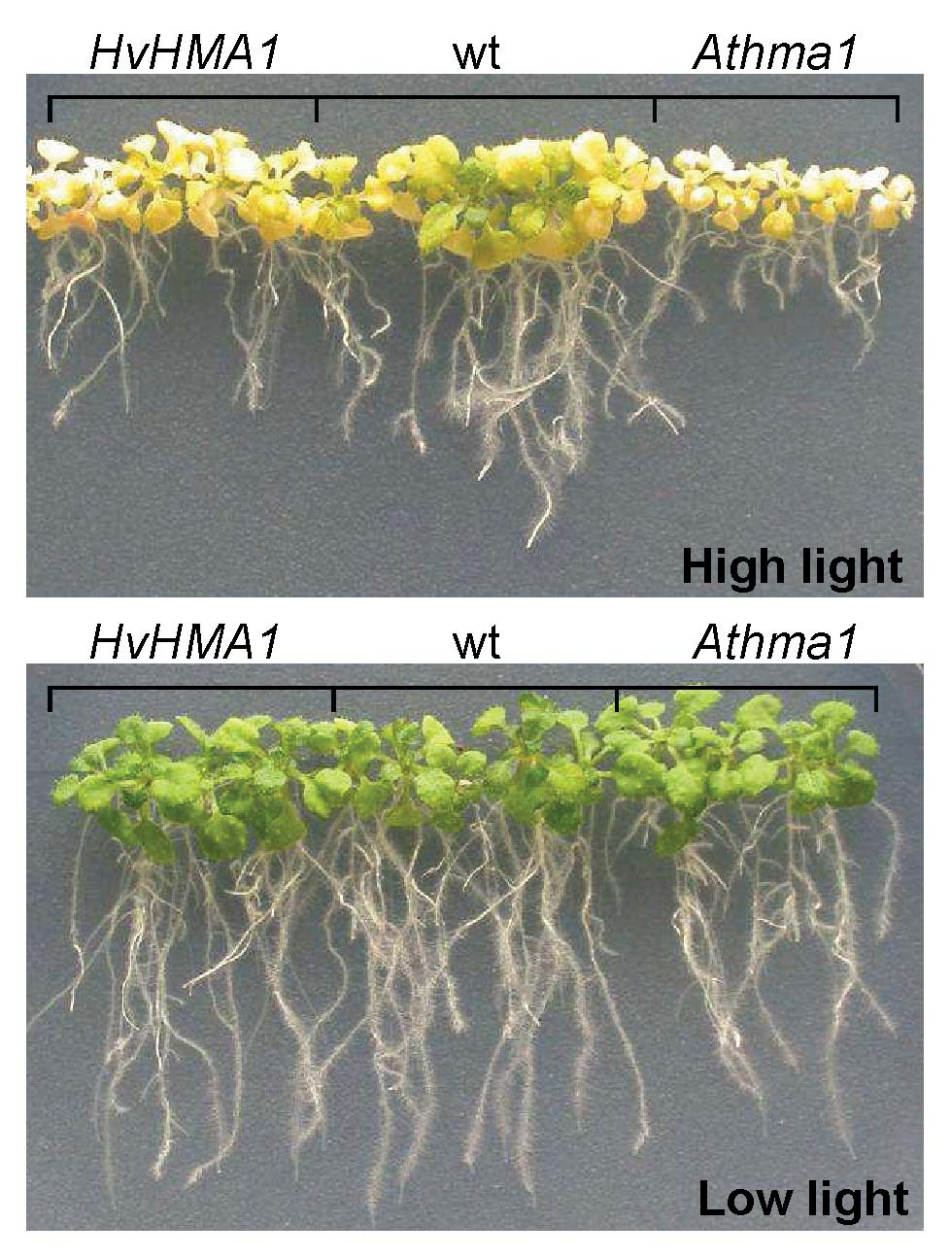
HvHMA1 partially rescues the high light induced phenotype of *Athma1* knockout plants. *A. thaliana Athma1::HvHMA1* (*HvHMA1*), wild-type (wt) and *Athma1* plants grown under normal and high light conditions.

**Figure 6:**
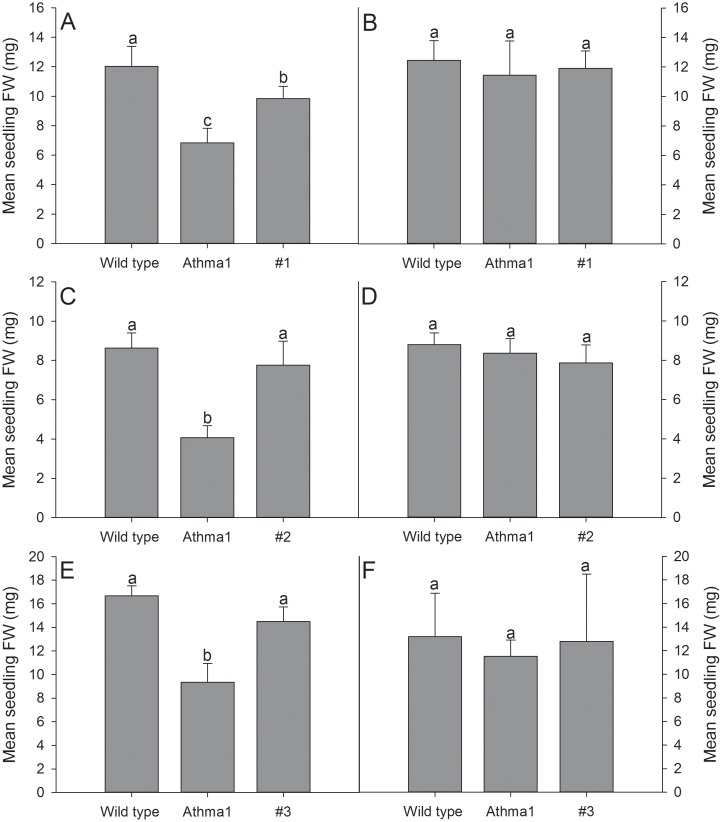
HvHMA1 rescues the decreased fresh weight of *Athma1* plants under high light conditions. Homozygous *Athma1::HvHMA1* lines (#1–3), wild-type (wt) and *Athma1* plants were grown under A), C) and E) high light and B), D) and F) normal light conditions. Plant fresh weight, of *HvHMA1* expressing plants, was significantly increased compared to *Athma1* plants under high light conditions and in most experiments the weight was not significantly different from wild-type, suggesting fresh weight rescue of *Athma1* by *HvHMA1* under high light conditions. B), D) and F) no significant differences were observed under normal light conditions. Data are the means ± SE (*n* = 5). Values with the same letter between lines within the same light treatment are not significantly different (P>0.05).

**Figure 7:**
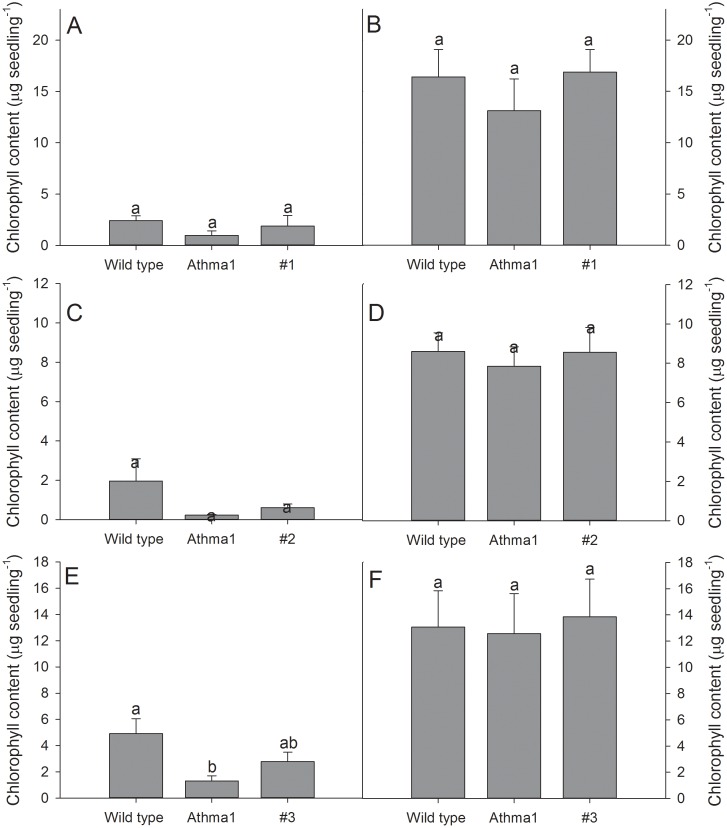
HvHMA1 only partially rescues the chlorophyll content of *Athma1* plants under high light conditions. Chlorophyll content was not significantly increased in *Athma1::HvHMA1* plant lines (#1–3) compared to *Athma1* plants in A), C) and E) high light, while it was still significantly lower than wild-type, suggesting partial rescue of chlorophyll content by HvHMA1. B), D) and F) no significant differences were observed under normal light conditions. Data are the means ± SE (*n* = 5). Values with the same letter between lines within the same light treatment are not significantly different (P>0.05).

As HvHMA1 is closely related to AtHMA1 it is possible that they have similar functions. To test this hypothesis, we expressed *HvHMA1* in *Athma1* knockout mutants to determine whether phenotypes displayed by *Athma1* mutants could be rescued by *HvHMA1*. *Athma1-1* and *Athma1-2* mutant plants were isolated independently but are identical to those described in Kim *et al.* (2009) [43]. However, they are different to the *Athma1* mutants characterized by Seigneurin-Berny *et al.* (2006) [44]. As the latter mutants showed a light-sensitive phenotype, we tested *Athma1-1* and *Athma1-2* under high (300 µmol m^−2^ s^−1^) and low light (72 µmol m^−2^ s^−1^) conditions. Seedling fresh weight as well as chlorophyll levels were significantly reduced in both mutants compared to wild-type under high light conditions but were similar to wild-type under low light conditions (Figure 5, 6 and 7) indicating high-light photosensitivity of *Athma1-1* and *Athma1-2*.

Six transgenic *Athma1-2* lines expressing *HvHMA1* under control of the 35S promoter were isolated (Figure S3). Three of the lines were tested for their photosensitivity and compared to wild-type and the *Athma1-2* mutant. Under high light, lines expressing *HvHMA1* showed a significantly greater fresh weight than the *Athma1-2* mutant with a similar value to wild-type plants indicating that *HvHMA1* has restored the fresh weight defect (Figure 5 and 6) whereas expression of *HvHMA1* in *Athma1-2* did not restore chlorophyll levels fully (Figure 5 and 7). We conclude that *HvHMA1* partially complements a mutation in its orthologue *AtHMA1*.

### A Thapsigargin-sensitive ATPase Promotes Export of Zn from Barley Chloroplasts

AtHMA1 has previously been implicated in both import of Cu [44] and export of Zn from chloroplasts [43]. We isolated intact chloroplasts from barley in order to study their ability to import or export Zn and Cu in an Mg-ATP dependent manner. Thapsigargin is a specific inhibitor of P_2A_ Ca^2+^ pumps [69] that are absent from chloroplasts [36]. However, as the HvHMA1 sequence contains a conserved thapsigargin-binding motif (see above), and since thapsigargin has previously been shown to inhibit AtHMA1 containing a similar motif [45], we employed thapsigargin as a pharmacological tool to potentially inhibit activity of HvHMA1 in chloroplasts.

**Figure 8:**
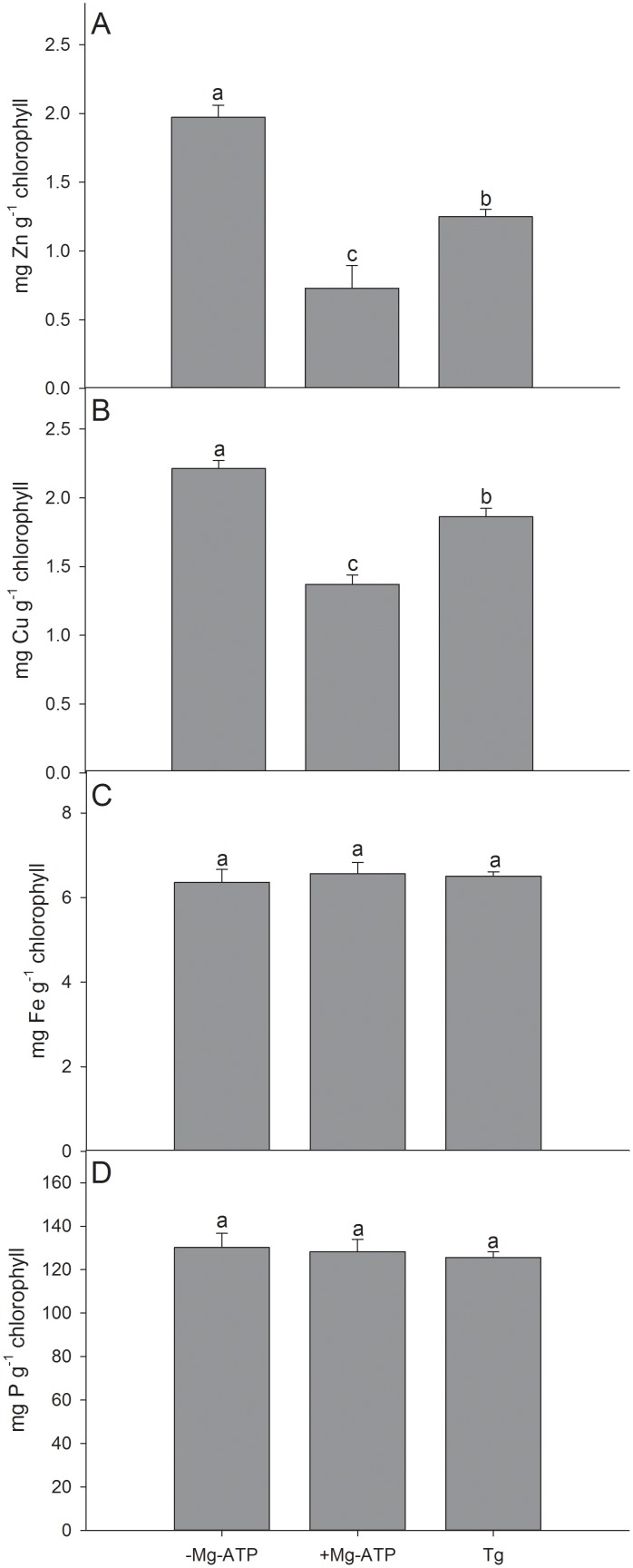
Zn and Cu efflux from barley chloroplasts is ATP dependent and inhibited by Thapsigargin. Metal transport assay on purified chloroplasts showed significantly reduced A) Zn and B) Cu content after addition of Mg-ATP, suggesting Mg-ATP induced Zn and Cu export. After addition of Thapsigargin (Tg) Zn and Cu content was again increased. Values with the same letter between treatments are not significantly different (P>0.05). Data are the means ± SE (*n* = 5).

Following addition of Zn, Cu, and Mg-ATP to isolated chloroplasts, the chloroplastic Zn and Cu content was significantly reduced (Figure 8A–B). When adding thapsigargin, the content of Zn and Cu was increased, although not completely restored (Figure 8A–B). This indicates that the observed export of Zn and Cu from chloroplasts is catalyzed by an ATP-driven, thapsigargin-sensitive transporter, which is likely to be HvHMA1. Transport of the Fe and P were not influenced by addition of ATP and thapsigargin (Figure 8C–D), which demonstrates that Zn and Cu export from chloroplasts were not the result of passive leaks. Taken together, the results suggest that in chloroplasts HvHMA1 is a thapsigargin-inhibited exporter of Zn and Cu.

As HvHMA1 is constitutively expressed, it might play an important role in regulation of Zn and Cu homeostasis in the chloroplast. Under conditions of Zn deficiency, there might be a need to mobilize chloroplastic Zn to redirect it to essential transcription factors and/or enzymes in the cytosol. During senescence in cereals, metals are believed to be translocated from the shoot to the grain, where at least Zn is known to ensure successful subsequent germination [21]. Under normal conditions, Zn and Cu are incorporated into Cu/Zn-SOD in the chloroplast, but under Zn deficiency, the expression and activity of Cu/Zn-SOD is down-regulated thereby releasing Zn [41]. The expression level of Cu/Zn-SOD is also down-regulated during Cu deficiency, but under these conditions Cu is thought to be remobilized to plastocyanin and is hence not exported from chloroplasts [14], [41]. This is in agreement with the observed lack of up-regulation of *HvHMA1* transcript level under Cu deficiency.

### Down-Regulation of *HvHMA1* by RNAi Causes Increased Zn and Cu Content in Grains

As our results indicated a role for HvHMA1 in heavy metal homeostasis in seeds,and leaves in particular under Zn deficiency, we decided to test this hypothesis by producing barley plants with an altered level of *HvHMA1* expression. For this purpose we attempted to produce plants over-expressing *HvHMA1* as well as plants in which expression of *HvHMA1* was reduced as a result of RNA interference.

A construct was made for over-expression of *HvHMA1* under control of the 2×35S promoter, which was transformed into more than a thousand embryos. Transformation procedures progressed as anticipated until rooting of plantlets was induced. Small roots developed but were quickly arrested in growth and subsequently plantlets died. Another over-expression construct was made with sequences encoding HvHMA1 fused to GFP with expression controlled by the 2×35S promoter. Also in this case plantlets died after setting small roots and no transgenic lines were obtained.

As an alternative strategy to investigate the effect of down-regulation of *HvHMA1* expression, barley was transformed with an *HvHMA1* RNA interference (RNAi) construct, under expression of the maize ubiquitin promoter. In order to identify and discard false positives, transformed plant lines were tested for insertion of the construct by RT-qPCR on genomic DNA from the T1 generation (Figure S4).

Positive plants were all down-regulated in *HvHMA1* expression compared to wild-type (Figure S5). Three T1 lines were selected for further analysis (# 17.5, 29.5, and 30.4) with *HvHMA1* expression levels of approximately 20%, 18%, and 15% respectively.

**Figure 9:**
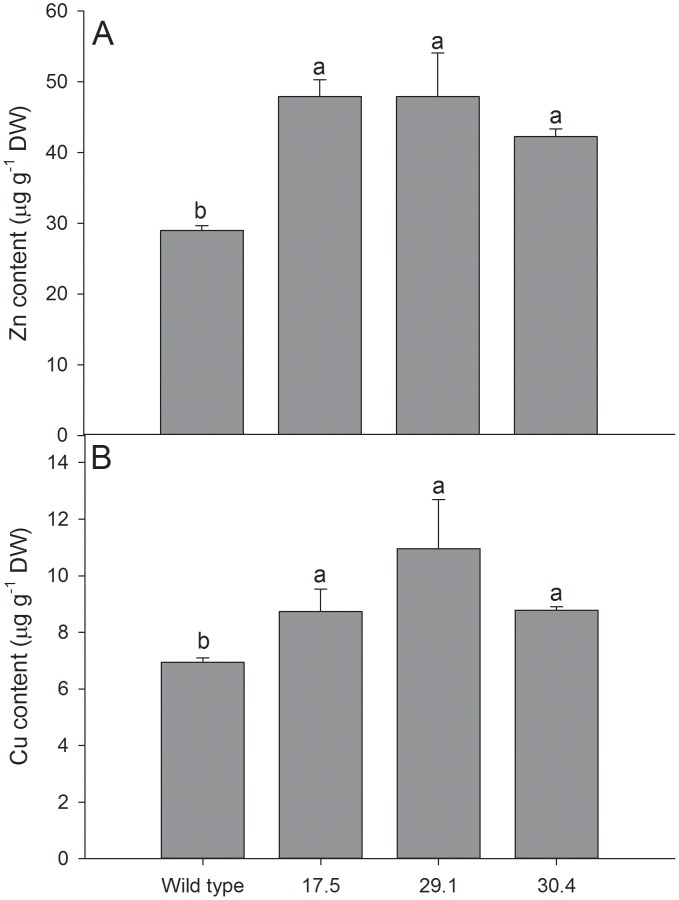
HvHMA1 is involved in Zn and Cu homeostasis in barley grains. Zn and Cu content was increased in grains from 3 different *HvHMA1* RNAi lines (lines 17.5, 29.5 and 30.4). A) Zn and B) Cu content was significantly increased in all tested grains compared to wild-type (wt). Values with the same letter between lines are not significantly different (P>0.05). Data are the means ± SE (*n* = 3).

Down-regulated plants showed no apparent phenotype when grown under normal greenhouse conditions in soil or in hydroponic cultures under high light conditions under Zn and Cu toxicity or deficiency. Elemental analysis of leaves was performed from plants grown in soil and in hydroponic cultures under the above mentioned stress conditions using ICP-OES. In leaves, no significant difference in elemental composition was found between wild-type and down-regulated plants. Purified chloroplasts isolated from RNAi plants had a tendency for higher Zn and Cu compared to wild-type chloroplasts (Figure S8). In grains of down-regulated plants grown in soil under greenhouse conditions, the contents of Zn and Cu were significantly increased compared to grains of wild-type and of segregating null mutants (Figure 9A–B and Figure S7). These findings indicate that HvHMA1 plays a significant role in accumulation of Zn and Cu in grains. The Zn and Cu content of grains is increased following knock-down of *HvHMA1* suggesting that HvHMA1 is somehow limiting the total Zn and Cu uptake of the grain. Down-regulation of HvHMA1 in aleurone cells may reduce cytoplasmic Zn and Cu and provide a sink for further uptake of these metals into the grain.

### HvHMA1 Shows Cu, Zn, Cd, Co, Mn, Ca and Fe Transport Activities Following Heterologous Expression in Yeast

To determine the substrate specificity of HvHMA1, we used metal-sensitive strains of the budding yeast *S. cerevisiae* for heterologous expression. For this purpose, HvHMA1 was expressed under the galactose inducible *GAL1* promoter in various mutant strains of *S. cerevisiae* affected in metal handling, including the Zn and Co sensitive *zrc1 cot1*, the Cu sensitive *ccc2*, the Cd sensitive *ycf1*, the Fe sensitive *ccc1* and the Mn sensitive *pmr1* strains. In addition we tested the function of HvHMA1 in Ca transport in the Ca dependent K616 strain. As a negative control, a non-functional mutant of HvHMA1 was constructed in which the essential aspartic acid residue was substituted by an asparagine residue (*Hvhma1*). *HvHMA1* was further expressed without the chloroplast targeting peptide (deletion of the first 50 residues; *Hvhma1Δ50*) and without the C-terminus including the His-stretch (deletion of 97 residues; *Hvhma1Δ97*). In AtHMA4 the extended C-terminal domain contains metal coordinating residues and when the Ct is expressed alone in yeast, it is able to confer Zn and Cd resistance to sensitive yeast strains by chelating excess heavy metals [31]. We therefore expressed the N-terminal domain alone (*Hvhma1Nt*) in the yeast strains to investigate its potential role in metal chelation.

**Figure 10:**
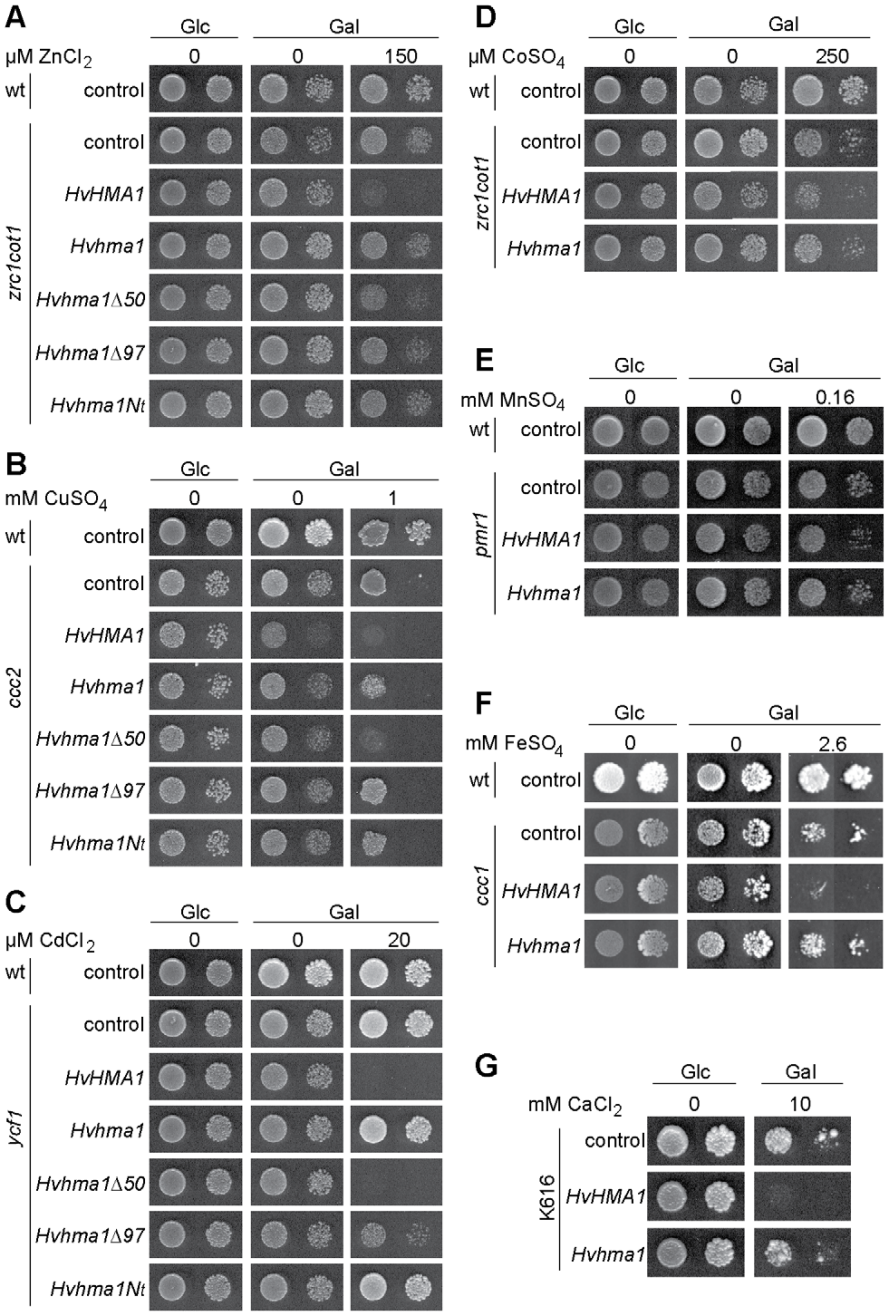
Expression of *HvHMA1* induced sensitivity to several divalent cations in mutant yeast strains. Expression of *HvHMA1* has a toxic effect on yeast growth compared to empty vector control (control), which was reversed when the pump is non-functional (*Hvhma1*). A) the Zn and Co sensitive yeast strain *zrc1cot1*, on 150 µM Zn, B) the Cu sensitive yeast strain *ccc2* on 1 mM Cu. C) the Cd sensitive yeast strain *ycf1* on 20 µM Cd, D) the Zn and Co sensitive yeast strain *zrc1cot1*, on 250 µM Co, E) the Fe sensitive strain *ccc2* on 2.6 mM Fe, F) Ca dependent yeast strain K616 on 10 mM Ca and G) the Mn sensitive *pmr1* yeast strain on 0.16 mM Mn. A), B), and C) When the N-terminus has been removed *Hvhma1Δ97* or when the *Hvhma1Nt* was introduced yeast growth was comparable to control. Expression of *Hvhma1Δ50* showed an intermediate growth of the yeast, between control and *HvHMA1*.

In all yeast strains, expression of *HvHMA1* resulted in yeast with increased metal sensitivity compared to control cells (Figure 10). All strains expressing the non-functional mutant Hvhma1D457N (*Hvhma1*) grew like the empty vector control, implying that the negative impact of growth is the result of an active pump and not an indirect result of heterologous expression (Figure 10) such as, e.g., metal chelation by the His-rich N-terminal part of the protein. The negative impact of HvHMA1 on metal homeostasis in yeast might result from increased cellular uptake of metals as was previously suggested for Athma1ΔN [43]. Removing N-terminal sequences, either the chloroplast targeting signal or the whole N-terminus, reduced the toxic effect of HvHMA1 on yeast growth (Figure 10). This implies that the N-terminal domain is important for enzyme activity or that its removal changes the expression level or the localisation of the pump in yeast. Expression of the His-rich N-terminal alone did not impact the Zn, Cu or Cd sensitivity of yeast compared to control cells. In conclusion, HvHMA1 is a broad specificity metal transporter that *in planta* may have other physiologically relevant roles in addition to transporting Zn and Cu.

## Conclusions

In the present study, we have characterized HvHMA1, a heavy metal ATPase of the cereal *H. vulgare*. Sequence analysis and complementation studies indicate that HvHMA1 is structurally and functionally equivalent to AtHMA1 of *A. thaliana*. We provide evidence that this ATPase is a broad specificity transporter localized to chloroplasts of leaves and to organellar structures in the endosperm of grains of barley. Our data suggests a role of HvHMA1 in exporting Zn and Cu from plastids. Such an activity could be important for redistribution of heavy metals within and between cells in response to changes in cellular demand, such as during Zn deficiency and during the process of grain filling.

## Supporting Information

Figure S1 **Alignment of HvHMA1, AtHMA1, OsHMA1 and AtHMA7.** The alignment reveals 8 putative TM segments in *HvHMA1* (underlined in Figure). Due to deletion of the residues separating TM segment 3 and 4 in *HMA1* sequences compared to *AtHMA7*, we predict these two TM segments to be situated in the membrane making a hairpin structure. Conserved motifs are highlighted, including the transduction motif (in red), the putative Tg binding motif (in green), the CPx/SPC motif (in yellow), the phosphorylation motif (in blue), ATP-binding motif (in pink) and HEGG motif (in grey).
(DOCX)

Figure S2 **Lambda scans from the cells shown in** **Figure 2** **.** The scans show GFP fluorescence in transgenic plants compared to no GFP fluorescence in wild-type plants. A) shows scans from wild type (Figure 2A) and transgenic barley (Figure 2D) leaf cells respectively, while B) shows scans from wild type (Figure 2G) and transgenic barley (Figure 2J) aleurone layer cells from grains respectively.
(TIF)

Figure S3 **Verification of** ***HvHMA1*** **expression in** ***A. thaliana Athma1*** **knockout plants.** RT-PCR on wild-type (wt), *Athma1* (hma1) and *HvHMA1::Athma1* (35S:HvHMA1 in *hma1* lines #1–6) plants showing expression of *HvHMA1* only in *Athma1::HvHMA1* plant lines. Actin expression was used as reference.
(TIF)

Figure S4 V**erification of positive** ***HvHMA1*** **RNAi** ***H. vulgare*** **lines.** Quantitative PCR on genomic DNA from *HvHMA1* RNAi transgenic barley plants using ubiquitin promoter specific primers. The relative number of PCR cycles to obtain a product was low for positive transgenic lines, while high for non-transgenic lines. GFP is a transgenic plant line used as positive control. Error bars indicate ±SE.
(TIF)

Figure S5 ***HvHMA1*** **expression was down-regulated in** ***HvHMA1*** **RNAi** ***H. vulgare*** **lines.** Several lines showed significant down-regulation to approximately 20% compared of wild-type level. Data is normalized to tubulin expression. Error bars indicate ±SE.
(TIF)

Figure S6 ***HvHMA1*** **DNA sequence used for creating the RNA interference construct.**
(DOCX)

Figure S7 **Zn and Cu grain content is comparable in wild type and segregating non-transgenic null-lines (Null) of** ***HvHMA1*** **RNAi plants.** A) Zn B) Cu content in grains from wild type vs. Null plants is not significantly different. Values with the same letter between lines are not significantly different (P>0.05). Data are the means ± SE (*n* = 3).
(TIF)

Figure S8 **Zn and Cu content of purified chloroplasts isolated from wildtype and** ***HvHMA1*** **RNAi plants (line 30.4).** Values with the same letter between lines are not significantly different (P>0.05). Data are the means ± SE (*n* = 2).
(TIF)

Table S1 **Oligonucleotide sequences used for cloning.**
(DOCX)

Table S2 **Yeast strains used for complementation studies.**
(DOCX)

Table S3 **Oligo sequences used for real-time PCR.**
(DOCX)
