# Supplementary material for: Barley HvHMA1 Is a Heavy Metal Pump Involved in Mobilizing Organellar Zn and Cu and Plays a Role in Metal Loading into Grains
Source: PLoS One. 2012 Nov 14;7(11):e49027. doi: 10.1371/journal.pone.0049027 (PMC3498361; doi:10.1371/journal.pone.0049027)
Supplement: Figure S1 — Alignment of HvHMA1, AtHMA1, OsHMA1 and AtHMA7. The alignment reveals 8 putative TM segments in HvHMA1 (underlined in Figure). Due to deletion of the residues separating TM segment 3 and 4 in HMA1 sequences compared to AtHMA7, we predict these two TM segments to be situated in the membrane making a hairpin structure. Conserved motifs are highlighted, including the transduction motif (in red), the putative Tg binding motif (in green), the CPx/SPC motif (in yellow), the phosphorylation motif (in blue), ATP-binding motif (in pink) and HEGG motif (in grey). (DOCX) [file pone.0049027.s001.docx]

**Figure S1**

Hvhma1 ------------------------------------------------------------

OsHma1 ------------------------------------------------------------

athma1 ------------------------------------------------------------

RAN1 MAPSRRDLQLTPVTGGSSSQISDMEEVGLLDSYHNEANADDILTKIEEGRDVSGLRKIQV 60

Hvhma1 ------------------------------------------------------------

OsHma1 ------------------------------------------------------------

athma1 ------------------------------------------------------------

RAN1 GVTGMTCAACSNSVEAALMNVNGVFKASVALLQNRADVVFDPNLVKEEDIKEAIEDAGFE 120

Hvhma1 MQFLTASAACSSSAPPLPRPAHLLRVSRPPPFPHLR----RRRAPHPPSVSLSLAPKPSL 56

OsHma1 MQLLTAASASASSAAASPPSAHLLRLSRPPPFPHLR----RRCAPHLPSKPLNLAARSPL 56

athma1 MEPATLTRSSSLTRFPYRRGLSTLRLARVNSFSILPPKTLLRQKPLRISASLNLPPRSIR 60

RAN1 AEILAEEQTQATLVGQFTIGGMTCAACVNSVEGILRDLPGVKRAVVALSTSLGEVEYDPN 180

Hvhma1 LAASRRSLLFTPRAHGDHHHHHHHHHHGHGHGHG-------------------------- 90

OsHma1 LLA-RRSLPFAPRAHGDHHHGHHHHHHGHGHSH--------------------------- 88

athma1 LRA-------VEDHHHDHHHDDEQDHHNHHHHHH-------------------------- 87

RAN1 VINKDDIVNAIEDAGFEGSLVQSNQQDKLVLRVDGILNELDAQVLEGILTRLNGVRQFRL 240

Hvhma1 ----------------HHGHGDDGVEVRGGGG-GAAVMRMARTIG---WADVADALREHL 130

OsHma1 ----------------HHGP-----EVHGSGG-GAAVMRVAKAIG---WADVADALREHL 123

athma1 ----------------QHGCCS--VELKAESKPQKMLFGFAKAIG---WVRLANYLREHL 126

RAN1 DRISGELEVVFDPEVVSSRSLVDGIEEDGFGKFKLRVMSPYERLSSKDTGEASNMFRRFI 300

Hvhma1 QLCCISLGLLLTAAVCPHVPLLNSVGRLPA-----------ALIAIAFPLVGVSAALDAL 179

OsHma1 QLCCISLGLLLIAAACPHIPVLNSVRRLQD-----------ALIAVAFPLVGVSAALDAL 172

athma1 HLCCSAAAMFLAAAVCPYLAPEPYIKSLQN-----------AFMIVGFPLVGVSASLDAL 175

RAN1 SSLVLSIPLFFIQVICPHIALFDALLVWRCGPFMMGDWLKWALVSVIQFVIGKRFYVAAW 360

Hvhma1 VDIADGKINIHVLMALAAFASIFMGN----------------SLEGGLLLAMFNLAHIAE 223

OsHma1 VNIADGKINIHVLMALAAFASIFMGN----------------SLEGGLLLAMFNLAHIAE 216

athma1 MDIAGGKVNIHVLMALAAFASVFMGN----------------ALEGGLLLAMFNLAHIAE 219

RAN1 RALRNGSTNMDVLVALGTSASYFYSVGALLYGAVTGFWSPTYFDASAMLITFVLLGKYLE 420

Hvhma1 EYFTSKSMYDVRELKENHPEFALLLETSGDESAHFSNLNYAKVPVHDLEVGSHILVRAGE 283

OsHma1 EHFTSKSMIDVRELKENHPEFALLLETCGDQSAQFANLCYTKVPVHDLEVGSHILVRAGE 276

athma1 EFFTSRSMVDVKELKESNPDSALLIEVHNGNVPNISDLSYKSVPVHSVEVGSYVLVGTGE 279

RAN1 SLAKGKTSDAMKKLVQLTPATAILLTEGKGG----KLVGEREIDALLIQPGDTLKVHPGA 476

Hvhma1 AVPVDGEVYQGSSTITIEHLTGETKPVERTVGDAIPGGARNLEGMMIVKVTKSWEDSTLN 343

OsHma1 AVPVDGEVYQGSSTVTIEHLTGETKPLERTVGDAIPGGARNLEGMMIVKVTKSWEDSTLN 336

athma1 IVPVDCEVYQGSATITIEHLTGEVKPLEAKAGDRVPGGARNLDGRMIVKATKAWNDSTLN 339

RAN1 KIPADGVVVWGSSYVNESMVTGESVPVSKEVDSPVIGGTINMHGALHMKATKVGSDAVLS 536

Hvhma1 RIVQLTEEGQLNKPKLQRWLDEFGEHYSKVVVALSLAVAL-------LGPFLFKWPFFGN 396

OsHma1 RIVQLTEEGQLNKPKLQRWLDEFGEHYSRVVVVLSLVVAL-------LGPLLFKWPFFGN 389

athma1 KIVQLTEEAHSNKPKLQRWLDEFGENYSKVVVVLSLAIAF-------LGPFLFKWPFLST 392

RAN1 QIISLVETAQMSKAPIQKFADYVASIFVPVVITLALFTLVGWSIGGAVGAYPDEWLPENG 596

Hvhma1 SVCRGSIYRGLGLMVAASPCALAVAP-LAYATAISSLASKGILLKGGHVLDALSSCQSIA 455

OsHma1 SVCRGSIYRGLGLMVAASPCALAVAP-LAYATAISSLASKGILLKGGHVLDALSACQSIA 448

athma1 AACRGSVYRALGLMVAASPCALAVAP-LAYATAISSCARKGILLKGAQVLDALASCHTIA 451

RAN1 THFVFSLMFSISVVVIACPCALGLATPTAVMVATGVGATNGVLIKGGDALEKAHKVKYVI 656

Hvhma1 FDKTGTLTTGKLMCKAIEPIHGHLDASNG-VDPSCCTPNCESEALAVAAAMEKGTTHPIG 514

OsHma1 FDKTGTLTTGKLMCKAIEPIHGHSDVTNDFSDQACCTPNCESEALAVAAAMEKGTTHPIG 508

athma1 FDKTGTLTTGGLTCKAIEPIYGHQGGTNS-SVITCCIPNCEKEALAVAAAMEKGTTHPIG 510

RAN1 FDKTGTLTQGKATVTTTK-VFSEMDRGEFLTLVASAEASSEHPLAKAIVAYARHFHFFDE 715

Hvhma1 RAVLKHSVGRDLP----VVAVESFESLPGRGVVATLSG-IKARDNESEFAKASIGSVEYI 569

OsHma1 RAVLDHSVGKDLP----LVAVESFECLPGRGVVATLSG-VKAGNNEDELSKASIGSVEYI 563

athma1 RAVVDHSVGKDLP----SIFVESFEYFPGRGLTATVNG-VKTVAEESRLRKASLGSIEFI 565

RAN1 STEDGETNNKDLQNSGWLLDTSDFSALPGKGIQCLVNEKMILVGNRKLMSENAINIPDHV 775

Hvhma1 SSLYRSYGESEQIKEAVKCSAFGPEFVQAALSVDKKVTLFHFEDEPRTGVCEVIYTLREK 629

OsHma1 SSLYRSSGESEQIKEAVKASAFGPEFVQAALTVDKKVTLFHFEDEPRSGVCEVISTLRDK 623

athma1 TSLFKSEDESKQIKDAVNASSYGKDFVHAALSVDQKVTLIHLEDQPRPGVSGVIAELKSW 625

RAN1 EKFVEDLEESGKTGVIVAYN-------------GKLVGVMGIADPLKREAALVVEGLLRM 822

Hvhma1 AKLRIMMLTGDHESSAQRVAKAVCIEEVHFSLKPEDKLNKVKAVSREGGGGLIMVGDGIN 689

OsHma1 AKLRIMMLTGDHESSALRVAKAVCIDEVHCCLKPEDKLNKVKAVSREGGGGLIMVGDGIN 683

athma1 ARLRVMMLTGDHDSSAWRVANAVGITEVYCNLKPEDKLNHVKNIAREAGGGLIMVGEGIN 685

RAN1 G-VRPIMVTGDNWRTARAVAKEVGIEDVRAEVMPAGKADVIRSLQKD-GSTVAMVGDGIN 880

Hvhma1 DAPALAAATVGIVLAQRASATAVAVADVLLLQDNLCVVPFCIAKARQTTSLVKQSVALAL 749

OsHma1 DAPALAAATVGIVLAQRASATAVAVADVLLLQDNICGVPFCIAKARQTTSLVKQSVALAL 743

athma1 DAPALAAATVGIVLAQRASATAIAVADILLLRDNITGVPFCVAKSRQTTSLVKQNVALAL 745

RAN1 DSPALAAADVGMAIG-AGTDVAIEAADYVLMRNNLEDVITAIDLSRKTLTRIRLNYVFAM 939

Hvhma1 TCIVFAALPSVLGFLPLWLTVLLHEGGTLLVCLNSIRALNPPTWSWADDIRQLVHSLKNY 809

OsHma1 SCIVFAALPSVLGFLPLWLTVLLHEGGTLLVCLNSIRALNSPTWSWVDDIRQLINSLRKY 803

athma1 TSIFLAALPSVLGFVPLWLTVLLHEGGTLLVCLNSVRGLNDPSWSWKQDIVHLINKLR-- 803

RAN1 AYNVVSIPIAAGVFFPVLRVQLPPWAAGACMALSSVSVVCSSLLLRRYKKPRLTTVLKIT 999

Hvhma1 VSAKLNSSSSDCSASTVPL 828

OsHma1 ISSKLQSTSSNYVVDAVPL 822

athma1 -SQEPTSSSSNSLSSAH-- 819

RAN1 TE----------------- 1001
