# Supplementary figures and images for: Barley HvHMA1 Is a Heavy Metal Pump Involved in Mobilizing Organellar Zn and Cu and Plays a Role in Metal Loading into Grains
Source: PLoS One. 2012 Nov 14;7(11):e49027. doi: 10.1371/journal.pone.0049027 (PMC3498361; doi:10.1371/journal.pone.0049027)

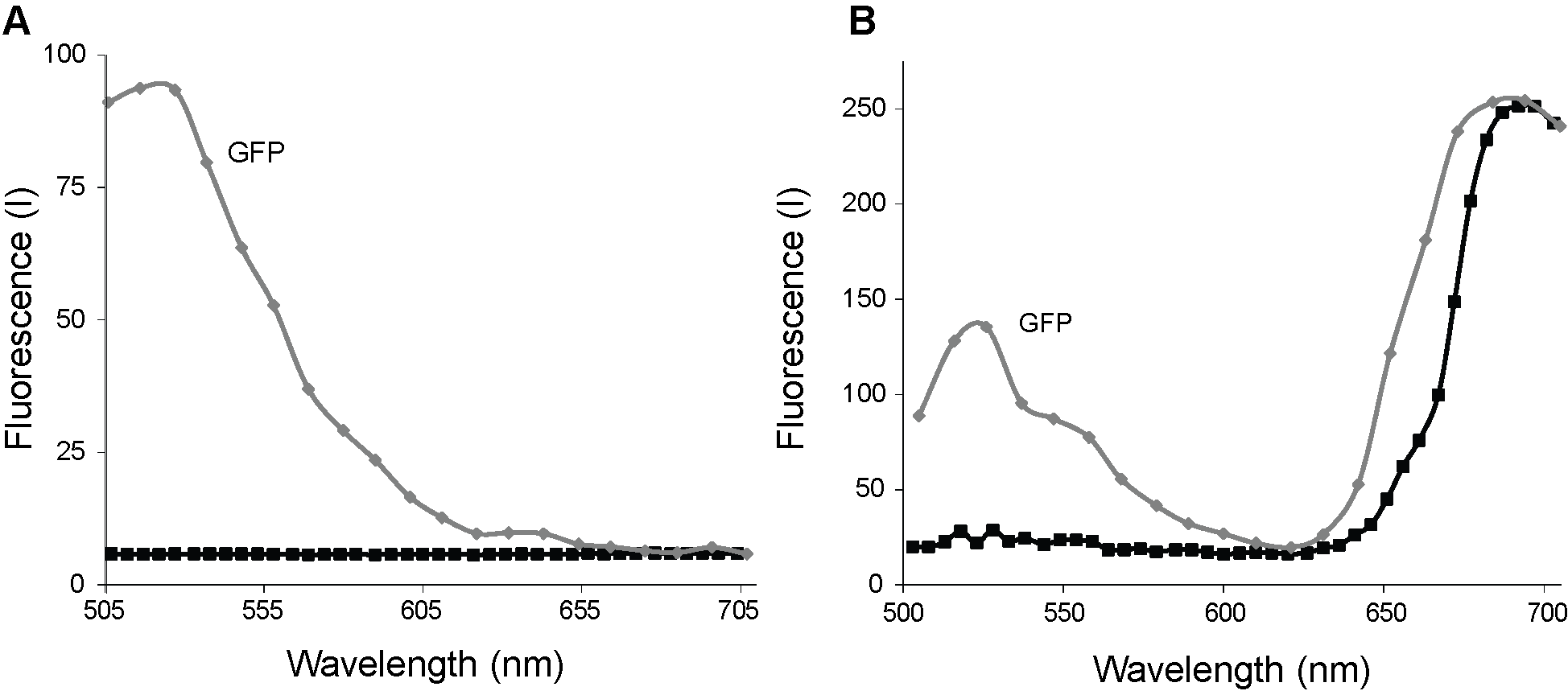

Supplement: Figure S2 — Lambda scans from the cells shown in Figure 2 . The scans show GFP fluorescence in transgenic plants compared to no GFP fluorescence in wild-type plants. A) shows scans from wild type (Figure 2A) and transgenic barley (Figure 2D) leaf cells respectively, while B) shows scans from wild type (Figure 2G) and transgenic barley (Figure 2J) aleurone layer cells from grains respectively. (TIF) [file pone.0049027.s002.tif]

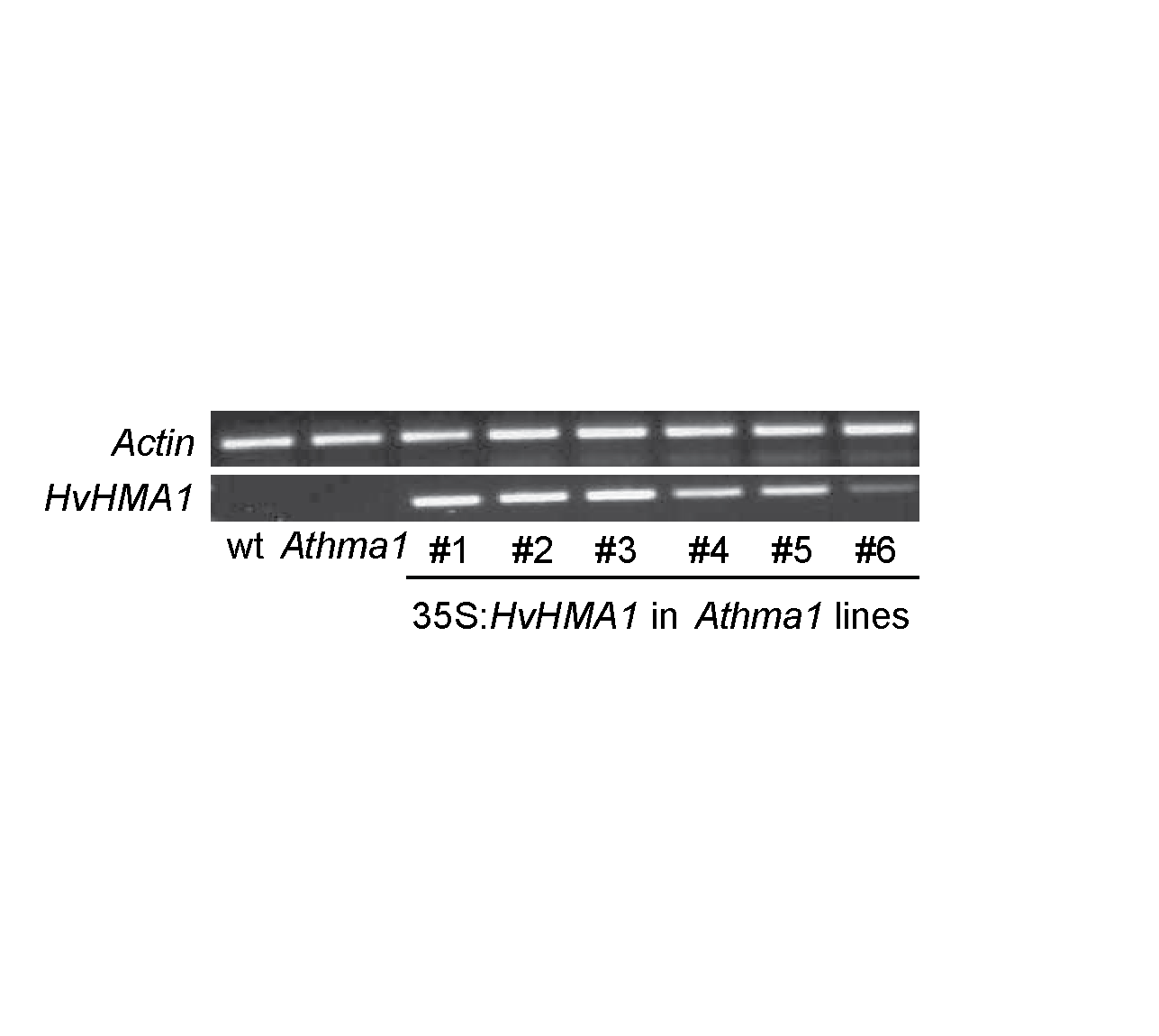

Supplement: Figure S3 — Verification of HvHMA1 expression in A. thaliana Athma1 knockout plants. RT-PCR on wild-type (wt), Athma1 (hma1) and HvHMA1::Athma1 (35S:HvHMA1 in hma1 lines #1–6) plants showing expression of HvHMA1 only in Athma1::HvHMA1 plant lines. Actin expression was used as reference. (TIF) [file pone.0049027.s003.tif]

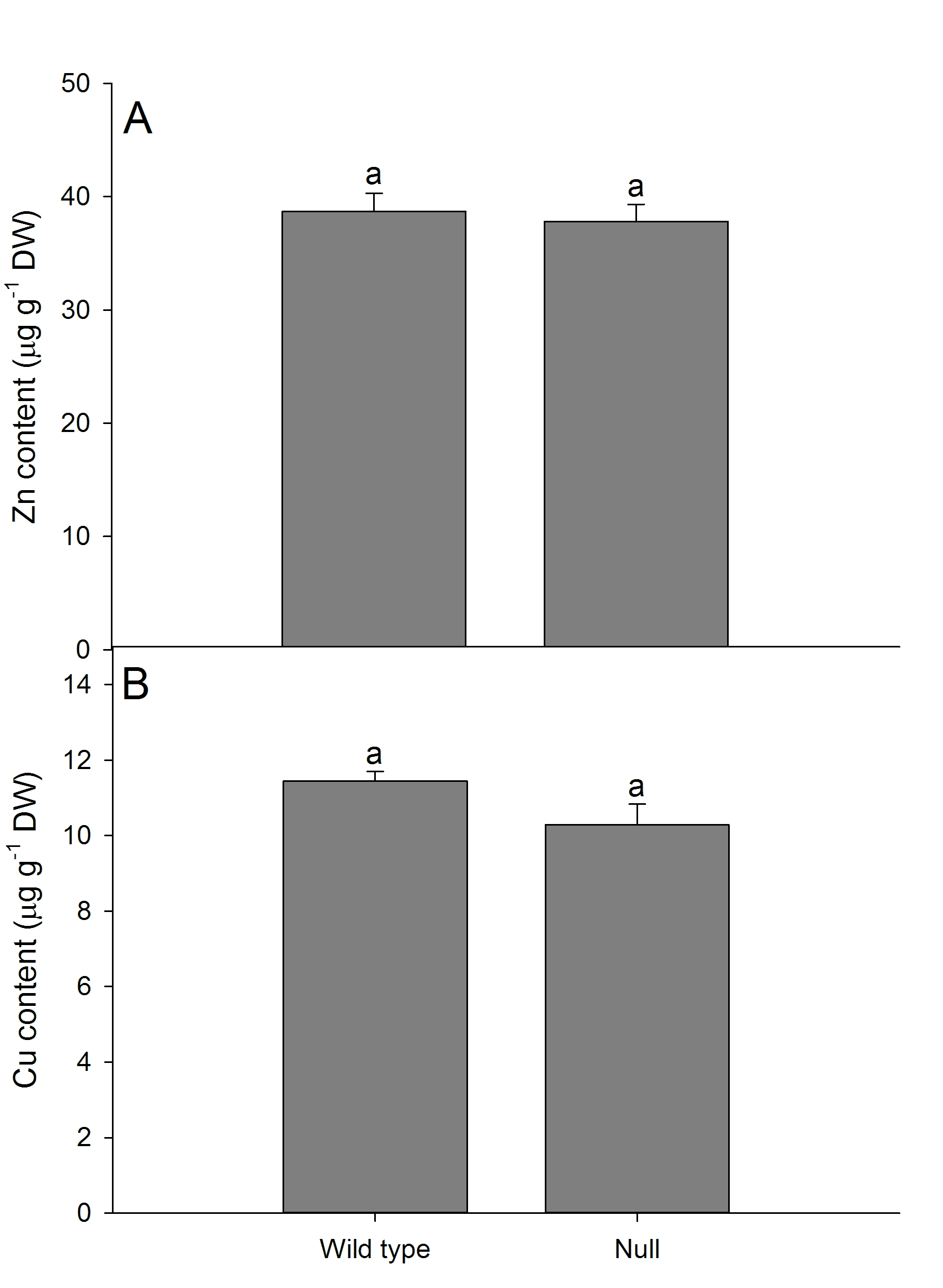

Supplement: Figure S7 — Zn and Cu grain content is comparable in wild type and segregating non-transgenic null-lines (Null) of HvHMA1 RNAi plants. A) Zn B) Cu content in grains from wild type vs. Null plants is not significantly different. Values with the same letter between lines are not significantly different (P>0.05). Data are the means ± SE (n = 3). (TIF) [file pone.0049027.s007.tif]

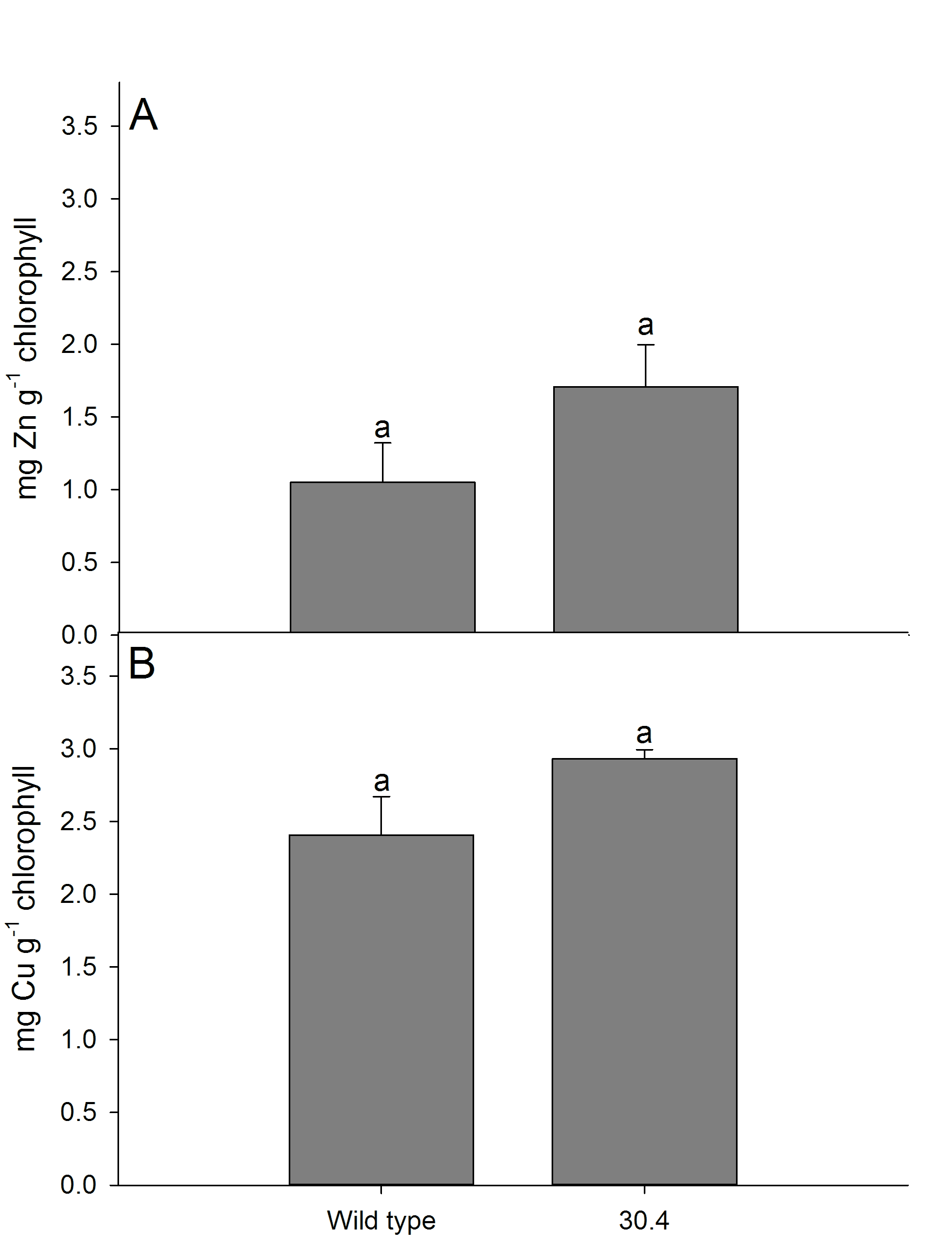

Supplement: Figure S8 — Zn and Cu content of purified chloroplasts isolated from wildtype and HvHMA1 RNAi plants (line 30.4). Values with the same letter between lines are not significantly different (P>0.05). Data are the means ± SE (n = 2). (TIF) [file pone.0049027.s008.tif]
