# Supplementary material for: Barley HvHMA1 Is a Heavy Metal Pump Involved in Mobilizing Organellar Zn and Cu and Plays a Role in Metal Loading into Grains
Source: PLoS One. 2012 Nov 14;7(11):e49027. doi: 10.1371/journal.pone.0049027 (PMC3498361; doi:10.1371/journal.pone.0049027)
Supplement: Figure S6 — HvHMA1 DNA sequence used for creating the RNA interference construct. (DOCX) [file pone.0049027.s006.docx]

**Figure S6**

1 TAGCACAACG CGCCAGTGCA ACAGCGGTAG CTGTTGCAGA TGTTCTGTTG

51 TTGCAGGATA ATTTATGTGT GGTGCCATTT TGTATCGCTA AAGCTCGTCA

101 AACAACTTCA TTGGTGAAGC AAAGCGTAGC TCTTGCCTTA ACCTGTATTG

151 TTTTTGCTGC ACTTCCTTCT GTCTTAGGAT TTCTTCCTCT TTGGTTGACA

201 GTTCTTCTCC ATGAAGGAGG AACCCTTCTC GTTTGCTTGA ACTCAATACG

251 AGCTC
