# Supplementary material for: Barley HvHMA1 Is a Heavy Metal Pump Involved in Mobilizing Organellar Zn and Cu and Plays a Role in Metal Loading into Grains
Source: PLoS One. 2012 Nov 14;7(11):e49027. doi: 10.1371/journal.pone.0049027 (PMC3498361; doi:10.1371/journal.pone.0049027)
Supplement: Table S1 — Oligonucleotide sequences used for cloning. (DOCX) [file pone.0049027.s009.docx]

**Table S1:** Oligonucleotide sequences used for cloning

| **Gene** | **Oligo sequence** |
| --- | --- |
| *HvHMA1* DNA | Forward 5’-GGGGGTACCCCACCATGCAGTTCCTCACCGCCTCC-3’ |
|  | Reverse 5’-CGGAATTCCTACAAAGGAACGGTGCTAGCTG-3’ |
|  | Forward 5’-GCTGAACTCAGTCGGGCGCCTGCC-3’ |
|  | Reverse 5’-GGCAGGCGCCCGACTGAGTTCAGC-3’ |
| *HvHMA1*  *promoter* | Forward 5’-GGGGGTACCGCTGTTGACTGGTTACAAGAGCC-3’ |
|  | Reverse 5’-CGGAATTCCGCTGCTCGCGGCGCGGCGCG-3’ |
| *GFP* | Forward 5’-TTAATTAAAGTAAAGGAGAAGAACTTTTC-3’ |
|  | Reverse 5’-GAGCTCTTAGTGGTGGTGGTGGTGGTG-3’ |
| *HvHMA1* RNAi | Forward 5’-CACCTAGCACAACGCGCCAGTGCAACAGCGGTAGCTGTTGC AGATGTTCTGTTGTTGCAGGATAATTTATGTGTGGTGCC-3’ |
|  | Reverse 5’-TTAAGGCAAGAGCTACGCTTTGCTTCACCAATGAAGTTGTTTGA  CGAGCTTTAGCGATACAAAATGGCACCACACATAAATTATCCTGC-3’ |
|  | Forward 5’- GAAGCAAAGCGTAGCTCTTGCCTTAACCTGTATTGTTTTTGC TGCACTTCCTTCTGTCTTAGGATTTCTTCCTCTTTGGTTGACAGTTCTTC-3’ |
|  | Reverse 5’-GAGCTCGTATTGAGTTCAAGCAAACGAGAAGGGTTCCTCCTT CATGGAGAAGAACTGTCAACCAAAGAGGAAG-3’ |
| *Hvhma1* | Forward 5’-CTTCTTGTCAATCGATTGCTTTTAACAAGACAGGCAC-3’ |
|  | Reverse 5’-GTGCCTGTCTTGTTAAAAGCAATCGATTGACAAGAAG–3’ |
| *Hvhma1Δ97* | Forward 5’-GGGGTACCATGGGGGTGGAGGTGCGCGGGGGA-3’ |
|  | Reverse 5’-GGAATTCCTACAAAGGAACGGTGCTAGCTGA-3’ |
| *Hvhma1Δ50* | Forward 5’-CCGGTACCATGGCCCCCAAACCCTCGCTCCTC-3’ |
|  | Reverse 5’-GGAATTCCTACAAAGGAACGGTGCTAGCTGA-3’ |
| *Hvhma1Nt* | Forward 5’-GGGGGTACCCCATGCAGTTCCTCACCGCCTCC-3’ |
|  | Reverse 5’-GGAATTCCTAGATCGTCCTCGCCATCCGCAT-3’ |
