# Supplementary material for: Barley HvHMA1 Is a Heavy Metal Pump Involved in Mobilizing Organellar Zn and Cu and Plays a Role in Metal Loading into Grains
Source: PLoS One. 2012 Nov 14;7(11):e49027. doi: 10.1371/journal.pone.0049027 (PMC3498361; doi:10.1371/journal.pone.0049027)
Supplement: Table S2 — Yeast strains used for complementation studies. (DOCX) [file pone.0049027.s010.docx]

**Table S2:** Yeast strains used for complementation studies

| **Mutant** | **Background** | **Mating-type** | **Genotype** | **Phenotype** | **Reference** |
| --- | --- | --- | --- | --- | --- |
| wt | BY4741 | MATa | *his3*Δ*1; leu2*Δ*0; met15*Δ*0; ura3*Δ*0* | wt | Euroscarf  Y00000 |
| *zrc1cot1* | BY4741 | MATa | *his3*Δ*1; leu2*Δ*0; met15*Δ*0; ura3*Δ*0; zrc1::natMX; cot1::kanMX4* | Zn and Co sensitive | (Dräger *et al*., 2004) |
| *ycf1* | BY4741 | MATa | *his3Δ1; leu2Δ0; met15Δ0; ura3Δ0;YDR135C::kanMX4* | Cd sensitive | Euroscarf Y04069 |
| *ccc2* | BY4741 | MATa | *his3∆1;leu2∆0;met15∆0;ura3∆0; YLR220w::kanMX4* | Cu sensitive | Euroscarf Y04169 |
| *ccc1* | BY4741 | MATa | *his3∆1;leu2∆0;met15∆0;ura3∆0; YLR220w::kanMX4* | Fe sensitive | Euroscarf Y04169* |
| *pmr1* | BY4741 | MATa | *his3∆1;leu2∆0;met15∆0;ura3∆0; YGL167c::kanMX4* | Mn sensitive | Euroscarf Y04534 |
| K616 | BY4741 | MATa | *pmr1::HIS3 pmc1::TRP1 cnb1::LEU2, ade2, ura3* | Ca dependent | (Cunningham and Fink, 1994) |

* The YCp-vector His 3 marker (Stratagene PRS-413) was transformed into the *ccc1* yeast strain achieving a yeast strain sensitive on high Fe levels [70].
