# Supplementary material for: Barley HvHMA1 Is a Heavy Metal Pump Involved in Mobilizing Organellar Zn and Cu and Plays a Role in Metal Loading into Grains
Source: PLoS One. 2012 Nov 14;7(11):e49027. doi: 10.1371/journal.pone.0049027 (PMC3498361; doi:10.1371/journal.pone.0049027)
Supplement: Table S3 — Oligo sequences used for real-time PCR. (DOCX) [file pone.0049027.s011.docx]

**Table S3:** Oligo sequences used for real-time PCR

| **Gene** | **Oligo sequence** |
| --- | --- |
| *HvHMA1* | Forward: 5’-AGCAGTTTCAAGGGAAGGAGGTG-3’ |
|  | Reverse: 5’-AACAACAGAACATCCGCAACAGC-3’ |
| *HvRNABP* | Forward: 5’-CGCCCAGTTATCCATCCATCTA-3’ |
|  | Reverse: 5’-AAAAACACCACAGGACCGGAC-3’ |
| *Ubiquitin promoter* | Forward: 5’-GACGGGATCGATTTCATGATTT-3’ |
|  | Reverse: 5’-AAACAAGTGCACGGCATATATTGA-3’ |
| *HvTUBA* | Forward: 5’-ACGATGGTGAGGATGGTGACG-3 |
|  | Reverse: 5’-TGGGCAGATCATAGGATAGCAGTAG-3’ |
| *HvActin* | Forward: 5’-TCGCTCCACCTGAGAGGAAG-3’ |
|  | Reverse: 5’-GCTAGGATGGACCCTCCGAT-3’ |
| *Cu/Zn-SOD* | Forward: 5’-CCCGTTGTAGGTCGCTGTTT-3’ |
|  | Reverse: 5’-GTAGCATGGCGACGGTAACA-3’ |
| *HvGAPDH* | Forward: 5’-GCTCAAGGGTATCATGGGTTACG-3’ |
|  | Reverse: 5’-GCAATTCCAGCCTTAGCATCAAAG-3’ |
| *AtActin2* | Forward: 5'-GGTAACATTGTGCTCAGTGGTGG-3’ |
|  | Reverse: 5'CTCGGCCTTGGAGATCCACATC-3’ |
| *HvHMA1*  *(Athma1 plants)* | Forward: 5'- TGTGTGGTGCCATTTTGTATC -3' |
|  | Reverse: 5'-ATGGACGAGTTGTCGGATG-3' |
